# Safety and efficacy of apatinib in patients with advanced gastric or gastroesophageal junction adenocarcinoma after the failure of two or more lines of chemotherapy (AHEAD): a prospective, single-arm, multicenter, phase IV study

**DOI:** 10.1186/s12916-023-02841-7

**Published:** 2023-05-05

**Authors:** Jin Li, Shukui Qin, Lu Wen, Junsheng Wang, Wenying Deng, Weijian Guo, Tongfu Jia, Da Jiang, Guifang Zhang, Yifu He, Yi Ba, Haijun Zhong, Lin Wang, Xiaoyan Lin, Jianwei Yang, Jun Zhao, Yuxian Bai, Xiangyuan Wu, Feng Gao, Guogui Sun, Yongjuan Wu, Feng Ye, Qiong Wang, Zhong Xie, Tienan Yi, Yong Huang, Guohua Yu, Lin Lu, Ying Yuan, Wei Li, Likun Liu, Yuping Sun, Ying Sun, Lifeng Yin, Zhiguo Hou

**Affiliations:** 1 Department of Oncology, Shanghai East Hospital, Tongji University School of Medicine, Shanghai, China; 2 Department of Oncology, Cancer Center of Jinling Hospital, Nanjing University of Chinese Medicine, No. 34 Biao, 34 Hao, Yanggongjing Road, Qinhuai District, Nanjing, 210002 Jiangsu Province China; 3 Department of Gastroenterology, Shanxi Provincial Cancer Hospital, Taiyuan, China; 4 Department of Internal Medicine, Anyang Cancer Hospital, Anyang, China; 5 Department of Gastroenterology, The Affiliated Cancer Hospital of Zhengzhou University & Henan Cancer Hospital, Zhengzhou, China; 6 Department of Medical Oncology, Fudan University Shanghai Cancer Center, Shanghai, China; 7 Department of Oncology, ZiBo Central Hospital, Zibo, China; 8 Department of Oncology, The Fourth Hospital of Hebei Medical University & Hebei Cancer Hospital, Shijiazhuang, China; 9 Department of Oncology, Xinxiang Central Hospital, Xinxiang, China; 10 Department of Oncology, The First Affiliated Hospital of USTC West District & Anhui Provincial Cancer Hospital, Hefei, China; 11 Department of Digestive Oncology, Tianjin Medical University Cancer Institute and Hospital, National Clinical Research Center for Cancer, Tianjin’s Clinical Research Center for Cancer, Key Laboratory of Cancer Prevention and Therapy, Tianjin, China; 12 Department of Medical Oncology, Zhejiang Cancer Hospital, Institute of Cancer and Basic Medicine, Chinese Academy of Sciences, Cancer Hospital of the University of Chinese Academy of Sciences, Hangzhou, China; 13 Department of Medical Oncology, Fujian Medical University Affiliated Union Hospital, Fuzhou, China; 14 Department of Abdominal Oncology, Fujian Medical University Cancer Hospital, Fujian Cancer Hospital, Fuzhou, China; 15 Department of Oncology, Changzhi People’s Hospital, Changzhi, China; 16 Department of Oncology, Harbin Medical University Cancer Hospital, Harbin, China; 17 Department of Oncology, The Third Affiliated Hospital of Sun Yat-Sen University, Guangzhou, China; 18 Department of Oncology, Heilongjiang Agricultural Reclamation Bureau General Hospital, Harbin, China; 19 Department of Radiotherapy and Chemotherapy, Tangshan People’s Hospital, Tangshan, China; 20 Department of Digestive Oncology, Baotou Tumor Hospital, Baotou, China; 21 Department of Oncology, The First Affiliated Hospital of Xiamen University, Xiamen, China; 22 Department of Oncology, Jiangyin People’s Hospital, Jiangyin, China; 23 Department of Oncology, The Affiliated Hospital of Guangdong Medical University, Zhanjiang, China; 24 Department of Oncology, Xiangyang Central Hospital, Affiliated Hospital of Hubei University of Arts and Science, Xiangyang, China; 25 Department of Oncology, The Second People’s Hospital of Hefei, Hefei, China; 26 Department of Oncology, Weifang People’s Hospital, Weifang, China; 27 Department of Oncology, 105 Hospital of People’s Liberation Army, Hefei, China; 28 Department of Oncology, The Second Affiliated Hospital Zhejiang University School of Medicine, Hangzhou, China; 29 Department of Oncology, The First Hospital of Jilin University, Changchun, China; 30 Department of Oncology, Shanxi Traditional Chinese Medical Hospital, Taiyuan, China; 31 Department of Oncology, Central Hospital Affiliated To Shandong First Medical University, Jinan, China; 32 Jiangsu Hengrui Pharmaceuticals Co., Ltd, Shanghai, China

**Keywords:** Advanced gastric cancer, Apatinib, Third- and later-line treatment, Phase IV study, Safety, Efficacy

## Abstract

**Background:**

Apatinib, a highly selective VEGFR2 inhibitor, significantly improved efficacy versus placebo as a third- and later-line treatment for advanced gastric cancer in phase 2 and 3 trials. This prospective, single-arm, multicenter phase IV AHEAD study was conducted to verify the safety and efficacy of apatinib in patients with advanced or metastatic gastric or gastroesophageal adenocarcinoma after at least two lines of systematic therapy in clinical practice settings.

**Methods:**

Patients with advanced gastric cancer who had previously failed at least two lines of chemotherapy received oral apatinib until disease progression, death or unacceptable toxicity. The primary endpoint was safety. The secondary endpoints included objective response rate (ORR), disease control rate (DCR), progression-free survival (PFS) and overall survival (OS). Adverse events were summarized by the incidence rate. Median OS and PFS were estimated using the Kaplan–Meier method. ORR, DCR, OS at 3 and 6 months, and PFS at 3 and 6 months were calculated, and their 95% CIs were estimated according to the Clopper-Pearson method.

**Results:**

Between May 2015 and November 2019, a total of 2004 patients were enrolled, and 1999 patients who received at least one dose of apatinib were assessed for safety. In the safety population, 87.9% of patients experienced treatment-related adverse events (TRAEs), with the most common hypertension (45.2%), proteinuria (26.5%), and white blood cell count decreased (25.3%). Additionally, 51% of patients experienced grade ≥ 3 TRAEs. Fatal TRAEs occurred in 57 (2.9%) patients. No new safety concerns were reported. Among the 2004 patients included in the intention-to-treat population, the ORR was 4.4% (95% CI, 3.6–5.4%), and DCR was 35.8% (95% CI, 33.7–38.0%). The median PFS was 2.7 months (95% CI 2.2–2.8), and the median OS was 5.8 months (95% CI 5.4–6.1).

**Conclusions:**

The findings in the AHEAD study confirmed the acceptable and manageable safety profile and clinical benefit of apatinib in patients with advanced gastric cancer as a third- or later-line of treatment.

**Trial registration:**

This study was registered with ClinicalTrials.gov NCT02426034. Registration date was April 24, 2015.

**Supplementary Information:**

The online version contains supplementary material available at 10.1186/s12916-023-02841-7.

## Background

Gastric cancer, one of the most common cancers worldwide, ranks fifth in incidence with an estimated 1,089,000 new cases and fourth in mortality with 769,000 deaths in 2020 [1]. And 403,000 new cases and 291,000 deaths are reported in China, according to the 2015 Chinese cancer registry data [2]. China has a large burden of gastric cancer worldwide, accounting for 37% and 38% of global incidence and mortality, respectively. The high mortality rate in China is associated with the high proportion of patients with advanced gastric cancer. Most patients are diagnosed at an advanced stage, with a median survival of less than one year [3].

Targeting angiogenesis has been validated as an effective way to combat tumor progression. Apatinib is a small-molecule tyrosine kinase inhibitor that highly selectively binds to and potently blocks vascular endothelial growth factor receptor 2 (VEGFR-2) [4]. In the randomized, placebo-controlled phase II and III trials, apatinib showed obvious antitumor activity in Chinese patients with advanced gastric or gastroesophageal junction adenocarcinoma as the third-line treatment or later [5, 6]. The phase II study demonstrated a more prolonged survival with apatinib at 850 mg once daily compared with 425 mg twice daily in 144 patients [5]; thus, apatinib at a dose of 850 mg once daily was recommended for the phase III trial. A significant improvement in overall survival (OS) as the primary endpoint was observed in patients treated with apatinib versus those treated with placebo (median OS, 6.5 vs 4.7 months) in the phase III study [6]. Additionally, phase II and phase III trials demonstrated an acceptable and manageable safety profile for apatinib. Hypertension, proteinuria, and hand-foot syndrome were the most common treatment-related adverse events (TRAEs) reported for apatinib in phase II and phase III trials [5, 6].

Apatinib was approved by the National Medical Products Administration as a third- or later-line treatment for advanced or metastatic gastric or gastroesophageal adenocarcinoma in China in October 2016. Currently, apatinib is the only tyrosine kinase inhibitor approved in China for gastric cancer patients who have received at least two lines of chemotherapy and is recommended by the Chinese Society of Clinical Oncology. Furthermore, the ATTRACTION-02 study showed that nivolumab improved OS in Asian patients with advanced gastric or gastroesophageal junction cancer compared with placebo as a third- or later-line treatment [7]. Accordingly, nivolumab was approved as a third- or later-line treatment for advanced or recurrent gastric cancer in Japan in 2017 and China in 2020. Disitamab vedotin (RC48) is an antibody–drug conjugate drug targeting HER2 developed in China. In a phase II study, disitamab vedotin achieved a clinically meaningful response and survival benefit for patients with previously heavily treated HER2-overexpressing gastric or gastroesophageal junction cancer [8]. The drug was therefore approved for the corresponding indication in China in 2021.Trifluridine/tipiracil is a compound drug. Trifluridine inhibits cell proliferation by interfering with DNA synthesis, and tipiracil increases exposure to trifluridine. As a result of the TAGS study, patients with heavily pretreated metastatic gastric cancer who received trifluridine/tipiracil had a longer OS than those who received a placebo [9]. The United States, Japan, and the European Union, but not China, approve Trifluridine/tipiracil to treat patients with metastatic gastric cancer who have already received at least two prior systemic treatments. Based on the findings of the DESTINY-Gastric01 study [10], the FDA approved trastuzumab deruxtecan for patients with locally advanced or metastatic HER2-positive gastric or gastroesophageal adenocarcinomas who have received a prior trastuzumab-based regimen in 2021.

Apatinib is the first treatment option approved in China for patients with heavily pretreated advanced gastric cancer. However, data from the phase II and phase III trials may not fully represent the safety and efficacy profiles of the drug in real-world clinical settings. Phase IV studies, with the advantage of a broad population and proximity to clinical practice, could provide more comprehensive information on licensed agents. Here we report a large-scale, prospective, multicenter study (AHEAD) to verify the safety and efficacy of apatinib in patients with advanced or metastatic gastric or gastroesophageal adenocarcinoma after at least two lines of chemotherapy.

## Methods

### Study design and participants

AHEAD was a prospective, single-arm, multicenter phase IV study which recruited participants from 150 sites in China. Eligible patients had an age between 18 and 75 years old; histologically or cytologically confirmed advanced gastric cancer or gastroesophageal junction adenocarcinoma with an extragastric measurable lesion per RECIST 1.1; an Eastern Cooperative Oncology Group (ECOG) performance status (PS) of 0–2; adequate hematological and hepatic function; failure of at least two lines of chemotherapy. Additionally, patients with observable lesions, but not measurable, could be enrolled according to the judgment of the investigators at each site. Patients at risk of gastrointestinal hemorrhage, such as a history of hematemesis and melena within three months before enrollment, active ulceration combined with a positive fecal occult blood test (+ +), were excluded from the present study. Other exclusion criteria were uncontrolled hypertension, coronary heart disease, arrhythmia (including men with QTc interval > 450 ms or women with QTc interval > 470 ms), cardiac insufficiency, coagulation abnormalities, and evidence of brain metastases. The study was conducted in accordance with international harmonized guidelines for good clinical practice and the principles of the Helsinki Declaration.

### Procedures

The recommended initial dose of apatinib was 850 mg once daily in 28-day cycles. However, the starting dose was chosen by the investigator based on the patient’s condition. Dose interruption and dose reduction were allowed according to the product label. Treatment continued until disease progression, intolerable toxicity, withdrawal of informed consent, or at the investigators’ discretion. Patients who had progression continued apatinib therapy at the discretion of the investigators.

### Endpoints

The primary endpoint was safety, assessed by the incidence and severity of adverse events, especially TRAEs. Adverse events of special interest were evaluated, including hypertension, proteinuria, hand-foot syndrome, bleeding events, hepatotoxicity, and cardiac toxicity. All adverse events were codified and summarized per the Medical Dictionary for Regular Activities (MedDRA) version 22.0 and graded according to the National Cancer Institute Common Terminology Criteria Adverse Events (NCI-CTCAE) version 4.0 up to 30 days after the last dose of apatinib.

The secondary endpoint was efficacy, including OS, progression-free survival (PFS), objective response rate (ORR), and disease control rate (DCR). OS was defined as the interval between enrollment and death from any cause or data censoring. PFS was defined as the interval between enrollment and tumor progression or death from any cause or data censoring. Tumor response was assessed per Response Evaluation Criteria in Solid Tumors (RECIST) version 1.1 by investigators.

### Statistical analyses

According to the requirements of the Chinese National Medical Products Administration for a post-marketing study, 2000 patients were planned to be enrolled in the study. The intention-to-treat (ITT) population was defined as all enrolled patients. The analyses of demographics and baseline characteristics were based on the ITT population. Efficacy was evaluated in the ITT population, and safety was assessed in those who received at least one dose of apatinib. Adverse events, TRAEs, as well as TRAEs of special interest, were summarized by the incidence rate. Median OS and PFS were estimated using the Kaplan–Meier method. ORR, DCR, OS at 3 and 6 months, and PFS at 3 and 6 months were calculated, and their 95% CIs were estimated according to the Clopper-Pearson method. A study steering committee was established to review the efficacy and safety data. Statistical analysis system version 9.4 was used for all analyses.

## Results

### Patient and treatment

**Fig. 1:**
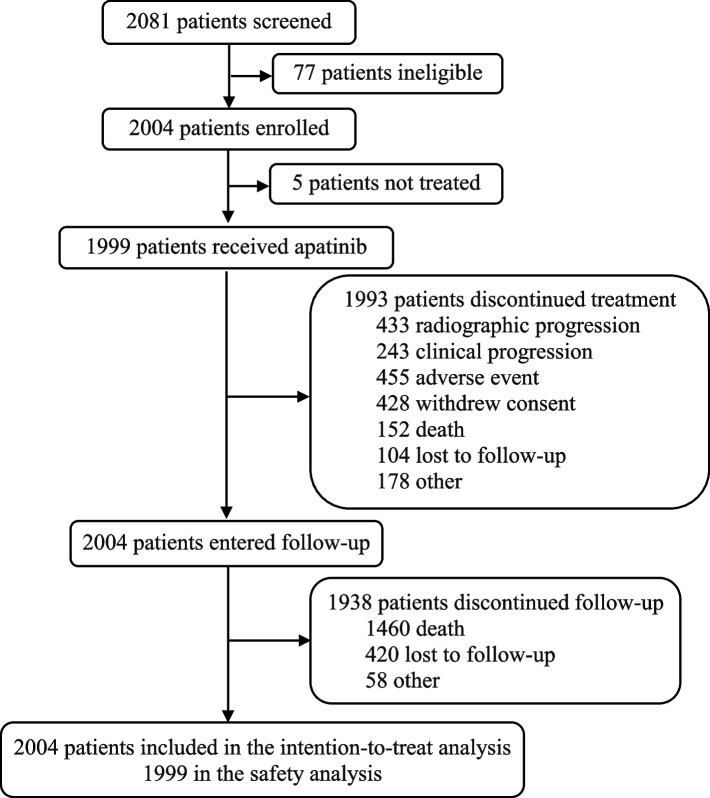
Study flowchart

Between May 2015 and November 2019, 2081 patients were screened, of whom 2004 were enrolled and included in the ITT set (Fig. 1). At the final data cutoff (May 29, 2020), 1999 patients received apatinib and were assessed for safety. An ITT population of 2004 patients was evaluated for efficacy. Table 1 shows the demographic and baseline characteristics of enrolled patients.

**Table 1:** Patient demographics and characteristics at baseline

Characteristic	ITT population (*n* = 2004)
**Age (years)**	
Median age	59
Range	19–85
**Gender, *n* (%)**	
Male	1438 (72%)
Female	566 (28%)
**ECOG performance status, *n* (%)**	
0	309 (15%)
1	1379 (69%)
2	308 (15%)
3	2 (< 1%)
Unknown	6 (< 1%)
**Stage, *n* (%)**	
III	70 (3%)
IV	1931 (96%)
Unknown	3 (< 1%)
**Extra-gastric metastases, *n* (%)**	
Yes	1979 (99%)
No	22 (1%)
Unknown	3 (< 1%)
**Number of metastatic sites, *n* (%)**	
≤ 2	1293 (65%)
> 2	686 (34%)
**Prior lines of chemotherapy, *n* (%)**	
≤ 2	1514 (76%)
> 2	486 (24%)
Unknown	4 (< 1%)
**Prior gastrectomy, *n* (%)**	
Yes	1491 (74%)
No	510 (25%)
Unknown	3 (< 1%)
**Previous systemic anticancer agents**	
Fluoropyrimidine	1976 (97%)
Platinum	1884 (94%)
Taxane	1554 (78%)
Irinotecan	542 (27%)
Anthracyclines	125 (6%)
Anti-HER2 therapy	75 (4%)
Ramucirumab	9 (< 1%)
Immunotherapy (anti-PD-1 or anti-CTLA-4)	5 (< 1%)
Other	266 (13%)

*ITT* Intention-to-treat, *ECOG* Eastern Cooperative Oncology Group

In total, 1795 (90%) patients initiated apatinib treatment at the daily dose of 500 mg, as shown in Additional file 1: Table S1. The median duration of apatinib treatment was two cycles, and the median daily exposure dose was 500 mg. Additionally, the relative median dose intensity was 92.6%. During treatment, 857 (43%) patients required dose interruption, and 422 (21%) patients required dose reduction.

### Safety

In the safety population, 1901 (95%) patients reported adverse events. TRAEs were reported in 1757 (88%) patients, and 1019 (51%) patients experienced grade ≥ 3 TRAEs (Table 2). Two hundred fifty-nine (13%) patients were reported with serious adverse events of any grade that were deemed to be related to the drug according to the investigator’s assessment, and most treatment-related serious adverse events (TRSAEs) were summarized as gastrointestinal disorders (119 [6%]). Fatal TRAEs occurred in 57 (3%) patients, the most being gastrointestinal disorders (24 [1%]) (Additional file 1: Table S2). As judged by the steering committee, except for gastrointestinal hemorrhage, gastrointestinal perforation, and central nervous system bleeding, other deaths were unlikely to be related to the study treatment.

**Table 2:** Treatment-related adverse events that occurred in ≥ 5% of patients (*n* = 1999)

Treatment-related adverse events, *n* (%)	All Grade	Grade ≥ 3
Any event	1757 (88%)	1019 (51%)
Serious adverse event	259 (13%)	203 (10%)
Hypertension	903 (45%)	490 (25%)
Proteinuria	530 (27%)	77 (4%)
White blood cell count decreased	506 (25%)	35 (2%)
Fatigue	438 (22%)	41 (2%)
Platelet count decreased	417 (21%)	73 (4%)
Neutrophil count decreased	363 (18%)	54 (3%)
hand-foot syndrome	330 (17%)	62 (3%)
Positive fecal occult blood test	288 (14%)	11 (1%)
Decreased appetite	250 (13%)	22 (1%)
Diarrhea	247 (12%)	25 (1%)
Aspartate aminotransferase increased	247 (12%)	26 (1%)
Anemia	243 (12%)	56 (3%)
Emesis	225 (11%)	22 (1%)
Blood bilirubin increased	214 (11%)	33 (2%)
Nausea	212 (11%)	18 (1%)
Alanine aminotransferase increased	169 (8%)	20 (1%)
Urine protein present	163 (8%)	13 (1%)
Hepatic function abnormal	158 (8%)	39 (2%)
Gamma-glutamyltransferase increased	154 (8%)	60 (3%)
Abdominal pain	138 (7%)	24 (1%)
Blood alkaline phosphatase increased	134 (7%)	28 (1%)

The incidence of grade ≥ 3 TRAEs and TRSAEs was generally comparable between subgroups defined by baseline characteristics, but there was a slightly higher incidence of grade ≥ 3 TRAEs in patients > 65 years compared with those ≤ 65 years (56% [329/568] vs 49% [690/1413]) (Additional file 1: Table S3).

As shown in Table 3, 1485 (74%) patients reported TRAEs of special interest of any grade, and the two most common TRAEs of special interest were hypertension (980 [49%]) and proteinuria (685 [34%]). Hypertension was also the most common grade ≥ 3 TRAEs of special interest (525 [26%]), while proteinuria, hand-foot syndrome, bleeding, hepatotoxicity, and cardiac toxicity had a low incidence of grade ≥ 3 events (Table 3). Most TRAEs of special interest did not require permanent discontinuation or dose reduction, and more than half of TRAEs of special interest were resolved or improved (Table 4).

**Table 3:** Treatment-related adverse events of special interest in apatinib-exposed patients (*n* = 1999)

Treatment-related adverse events of special interest, *n* (%)	Grade 1	Grade 2	Grade 3	Grade 4	Grade 5	All Grade^b^	Grade ≥ 3^b^
**Any event**	211 (11%)	527 (26%)	883 (44%)	79 (4%)	57 (3%)	1485 (74%)	1019 (51%)
**Hypertension**^a^	127 (6%)	328 (16%)	520 (26%)	5 (< 1%)	0	980 (49%)	525 (26%)
Hypertension	113 (6%)	300 (15%)	487 (24%)	3 (< 1%)	0	903 (45%)	490 (25%)
**Proteinuria**^a^	126 (6%)	142 (7%)	61 (3%)	1 (< 1%)	0	685 (34%)	89 (4%)
Proteinuria	252 (13%)	201 (10%)	76 (4%)	1 (< 1%)	0	530 (27%)	77 (4%)
**Hand-foot syndrome**^a^	126 (6%)	142 (7%)	61 (3%)	1 (< 1%)	0	330 (17%)	62 (3%)
**Overall bleeding**^a^	338 (17%)	100 (5%)	64 (3%)	12 (1%)	26 (1%)	540 (27%)	102 (5%)
Gastrointestinal hemorrhage	56 (3%)	49 (2%)	37 (2%)	12 (1%)	23 (1%)	177 (9%)	72 (4%)
CNS bleeding	0	0	0	1 (< 1%)	2 (< 1%)	3 (< 1%)	3 (< 1%)
Laboratory abnormality	258 (13%)	43 (2%)	12 (1%)	1 (< 1%)	0	314 (16%)	13 (1%)
Fecal occult blood positive	238 (12%)	39 (2%)	11 (< 1%)	0	0	288 (14%)	11 (< 1%)
**Hepatotoxicity**^a^	295 (15%)	155 (8%)	126 (6%)	19 (1%)	5 (< 1%)	600 (30%)	150 (8%)
Laboratory abnormality	237 (12%)	117 (6%)	97 (5%)	9 (< 1%)	0	460 (23%)	106 (5%)
Alanine aminotransferase increase	115 (6%)	34 (2%)	20 (1%)	0	0	169 (8%)	20 (1%)
Aspartate aminotransferase increase	179 (9%)	42 (2%)	25 (1%)	1 (< 1%)	0	247 (12%)	26 (1%)
Serum bilirubin increase	122 (6%)	59 (3%)	28 (1%)	5 (< 1%)	0	214 (11%)	33 (2%)
Hepatobiliary disease	91 (5%)	52 (3%)	36 (2%)	11 (1%)	5 (< 1%)	195 (10%)	52 (3%)
Hepatic injury	76 (4%)	43 (2%)	30 (2%)	5 (< 1%)	4 (< 1%)	158 (8%)	39 (2%)
**Cardiac toxicity **^a^	31 (2%)	9 (1%)	7 (< 1%)	0	0	47 (2%)	7 (< 1%)

^a^Baskets of MedDRA version 22.0 adverse events preferred terms were used

^b^Patients could have more than one grade of the same event

**Table 4:** Treatment-related adverse events of special interest, by the outcome and action taken

Treatment-related adverse events of special interest	Number of events	Outcome, *n* (%)					Action taken, *n* (%)		
		**Resolved**	**Improved**	**Persistent**	**Led to death**	**Unknown**	**Temporary interruption**	**Dose reduction**	**Permanent discontinuation**
Hypertension	1582	895 (57%)	221 (14%)	449 (28%)	0	17 (1%)	84 (5%)	39 (2%)	15 (1%)
Proteinuria	933	477 (51%)	95 (10%)	353 (38%)	0	8 (1%)	112 (12%)	31 (3%)	22 (2%)
Hand-foot syndrome	400	205 (51%)	55 (14%)	139 (35%)	0	1 (< 1%)	98 (25%)	36 (9%)	15 (4%)
Bleeding	784	477 (61%)	39 (5%)	227 (29%)	27 (3%)	14 (2%)	75 (10%)	10 (1%)	83 (11%)
Hepatotoxicity	1464	616 (42%)	147 (10%)	658 (45%)	6 (< 1%)	37 (3%)	105 (7%)	21 (1%)	55 (4%)
Cardiac toxicity	56	36 (64%)	3 (5%)	17 (30%)	0	0	10 (18%)	0	2 (4%)

Percentages are based on the total number of events in each category

Twenty-three (1%) patients experienced grade 5 gastrointestinal hemorrhage. One patient reported that grade 4 gastrointestinal hemorrhage did not recover but died of hemorrhagic shock. Three patients (> 1%) reported central nervous system bleeding, one patient improved, and two patients died. Additionally, grade ≥ 3 hepatobiliary disease (mainly hepatic injury) was reported in 52 (3%) patients, resulting in five deaths.

### Efficacy

**Fig. 2:**
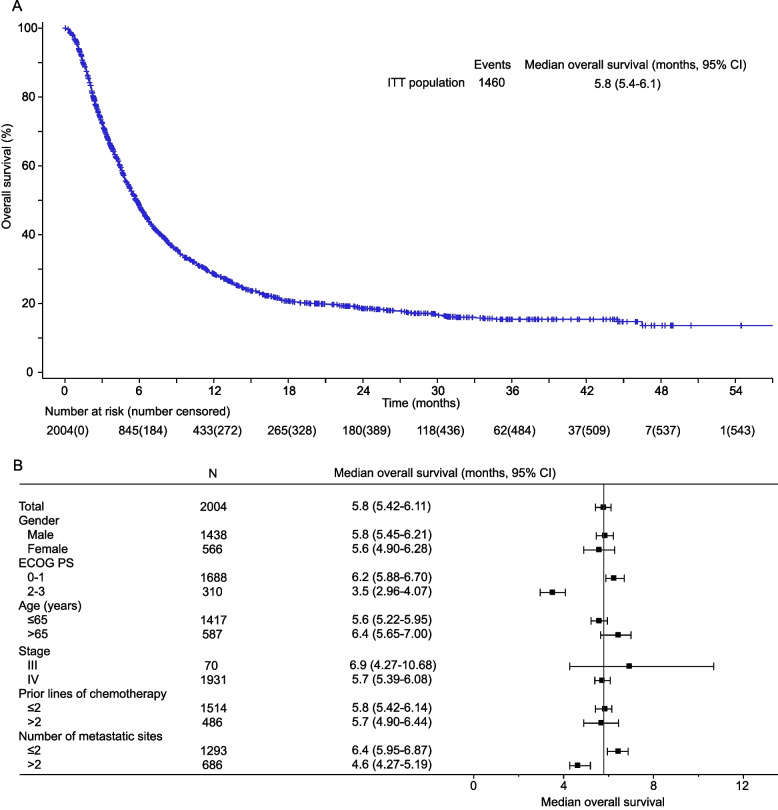
Kaplan–Meier analysis of overall survival (**A**) and subgroup analyses of overall survival (**B**) according to baseline characteristics in the intention-to-treat (ITT) population

In the ITT population, 1460 (73%) deaths occurred by the time of May 29, 2020, with a median follow-up time of 4.2 months. Median OS was 5.8 months (95% CI 5.4–6.1) (Fig. 2A), and OS at 3 and 6 months were 72.6% (95% CI 70.6–74.5) and 48.5% (95% CI 46.2–50.7), respectively. OS was generally similar across subgroups except for ECOG PS (0–1 vs 2–3; 6.2 vs 3.5 months) and the number of metastatic sites (≤ 2 vs > 2; 6.4 vs 4.6) (Fig. 2B).

At the time of the efficacy analysis, 1272 (63%) patients experienced disease progression or death. The median PFS was 2.7 months (95% CI 2.2–2.8), with PFS at 3 and 6 months of 42.6% (95% CI 40.0–45.2) and 20.4% (95% CI 18.2–22.7), respectively. Four patients achieved complete response, 85 patients achieved partial response, and 629 patients had stable disease, with an ORR of 4.4% (95% CI 3.6–5.4) and a DCR of 35.8% (95% CI 33.7–38.0). In order to determine the effect of apatinib on tumor size, the response was also evaluated in patients with target lesions at baseline, providing an ORR of 5.5% (95%CI 4.4–6.7) and a DCR of 38.3% (95% CI 35.9–40.7), respectively.

**Fig. 3:**
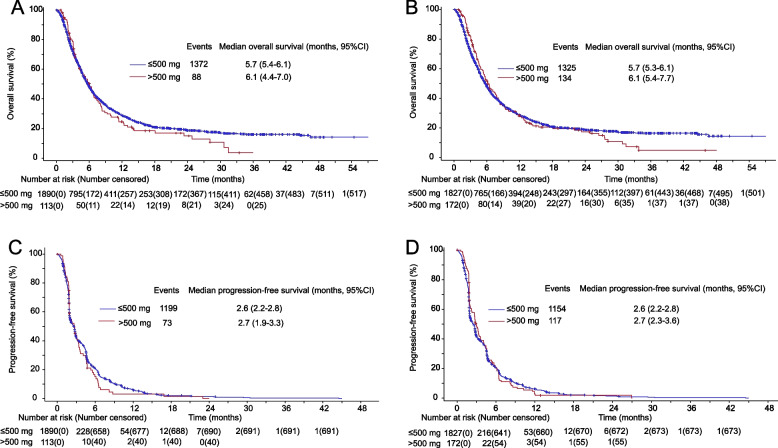
Subgroup analyses of overall survival according to initial dose (**A**) and daily exposure (**B**) of apatinib and progression-free survival according to initial dose (**C**) and daily exposure (**D**) of apatinib

Efficacy in terms of OS and PFS was generally comparable between patients with an initial dose of ≤ 500 mg and those with > 500 mg. Median OS was 5.7 months (95% CI 5.4–6.1) for ≤ 500 mg and 6.1 months (95% CI 4.4–7.0) for > 500 mg. Median PFS was 2.6 months (95% CI 2.2–2.8) for ≤ 500 mg and 2.7 months (95% CI 1.9–3.3) for > 500 mg. Meanwhile, the OS of patients with a daily exposure dose of ≤ 500 mg was almost similar to those with > 500 mg (Fig. 3).

## Discussion

The AHEAD study aimed to verify the safety and efficacy of third- or later-line apatinib in a broad population of patients with advanced gastric or gastroesophageal junction adenocarcinoma in China. Final data from this phase IV study demonstrated the well-established safety profile of apatinib and further confirmed the results of the phase III study.

The results of the double-blind, randomized, placebo-controlled phase III study supported apatinib's approval as a third- and later-line treatment for gastric or gastroesophageal junction adenocarcinomas in China. The study included patients between the ages of 18 and 70, with an ECOG PS of 0 to 1, with at least one measurable lesion per RECIST, and who had previously undergone two lines of chemotherapy. There was a significant improvement in OS and PFS with apatinib compared to placebo (median OS, 6.5 vs 4.7 months, hazard ratio = 0.709; median PFS, 2.6 vs 1.8 months, hazard ratio = 0.444). Apatinib showed an ORR of 2.84% and a DCR of 42.05% [6].

The AHEAD study set broad eligibility criteria that allowed enrollment of patients excluded from phase III studies, including elderly patients, patients with ECOG PS of 2, and patients with asymptomatic ascites and asymptomatic central nervous system metastases. Additionally, enrolled patients had a higher tumor burden than those in the phase III study of apatinib [6], such as 96% of patients with clinical IV staging and 34% of patients with metastatic lesions involving more than two organs. The study findings support that apatinib can bring clinical benefits to such patients without an increased risk of drug-related adverse events.

The overall toxicity profile in the AHEAD study was consistent with that in previous studies of apatinib [5, 6], with no new safety signals identified. The incidence of TRAEs and grade ≥ 3 TRAEs in the AHEAD study was similar to that reported in the phase III study of apatinib [6]. Furthermore, the incidence and severity of grade ≥ 3 TRAEs and TRSAEs did not vary broadly between patients with ECOG PS of 2–3 and those of 0–1, suggesting that apatinib was also tolerable in patients with poor ECOG PS.

Hypertension, proteinuria, and hand-foot syndrome are known adverse events associated with VEGF/VEGFR inhibition, frequently reported in studies of angiogenesis inhibitors such as bevacizumab [11], ramucirumab [12, 13], sorafenib [13], lenvatinib [14], and apatinib [6]. Interestingly, a previous study of post-hoc analyses of the phase III trial of apatinib in advanced gastric cancer demonstrated that the presence of hypertension, proteinuria, or hand-foot syndrome correlated with statistically significant and clinically meaningful outcomes [15]. Hypertension was the most common TRAEs in the AHEAD study. The grade ≥ 3 hypertension incidence was relatively higher in the AHEAD study than in the phase III study of apatinib (25% vs 5%). However, only 15 (1%) of hypertension events necessitated permanent discontinuation of apatinib, indicating that apatinib-related hypertension was well-controlled. Proteinuria may be caused by inhibiting the VEGF signaling pathway in pedal cells and mesangial cells in glomerular [16, 17] and represented the second most common TRAEs in the AHEAD study, with a low incidence of grade ≥ 3 proteinuria (4%). In 17% of patients, hand-foot syndrome occurred, but 3% reported grade ≥ 3 events. A low incidence of dose reduction or permanent discontinuation of apatinib was required to manage hand-foot syndrome in the AHEAD study. Furthermore, most hypertension events, proteinuria events, and hand-foot syndrome events recovered or improved, and none of the death occurred due to these events. The AHEAD data provide up-to-date evidence that the presence and development of hypertension, proteinuria, and hand-foot syndrome should not be considered barriers for patients with advanced gastric cancer when treated with apatinib.

Bleeding is considered a major safety concern for anti-VEGF/VEGFR agents [12, 18, 19]. In this trial, the majority of bleeding events were grade 1 to 2 laboratory abnormalities (15%). Clinically significant (grade ≥ 3) bleeding events occurred in 5% of patients, which was slightly higher than that reported in the previous trial of apatinib in advanced gastric cancer (3%) [6]. Among these bleeding events, 4% of patients had grade ≥ 3 gastrointestinal hemorrhage, and 23 (1%) patients reported grade 5 gastrointestinal hemorrhage. Apatinib was temporarily interrupted in 10% of bleeding events and permanently discontinued for 11%, suggesting most bleeding events were managed with the standard clinical procedure. Gastrointestinal hemorrhage is not unusually occurring in patients with advanced gastric cancer and may represent the progression of the disease. But these data remind us that a more cautious assessment for the risk of gastrointestinal hemorrhage should be performed in patients planning to receive apatinib, and more careful monitoring and immediate management are needed during apatinib treatment.

Patients with symptomatic brain metastases were excluded from this study. However, those with asymptomatic metastases could have been enrolled since brain imaging was not mandated before enrollment. In the AHEAD study, few central nervous system bleeding events were reported, suggesting that the advanced gastric cancer patients with asymptomatic brain metastasis treated with apatinib were well-tolerated.

Most hepatotoxicity events were grade 1 to 2 laboratory abnormalities. However, 3% of patients reported grade ≥ 3 hepatobiliary diseases, resulting in five deaths. Although the incidence of grade ≥ 3 hepatotoxicity was not high in the AHEAD study, changes in hepatic aminotransferase or serum bilirubin require regular monitoring in patients treated with apatinib to identify hepatic events early. Once physicians recognize hepatic events, in addition to immediate symptomatic therapy, they should take necessary procedures, including temporary interruption, dose reduction, or even discontinuation of apatinib, to prevent the development and deterioration of these events following the product introduction.

We also explored the effectiveness of apatinib in treating advanced gastric cancer in the AHEAD study. The median OS was 5.8 months, slightly shorter than that reported in a previous study (6.5 months) [6]. The efficacy in the AHEAD study might be potentially underestimated due to the inclusion of patients with ECOG PS of 2 and a higher rate of patients with more than two metastatic sites. The median OS in the AHEAD study was consistent with that of the TAGS study. A median OS of 5.7 months was achieved with trifluridine/tipiracil over 3.6 months with placebo (hazard ratio 0.69, 95% CI 0.56–0.85, *p* = 0.00058) [9]. Notably, the median OS of apatinib in the AHEAD study was slightly higher than that of nivolumab for advanced gastric cancer as a third-line treatment [20]. At the same time, the results should be interpreted cautiously since the head-to-head comparison was not performed.

Our data also allowed for the identification of patients with a better prognosis. OS was shorter in patients with ECOG PS of 2–3 versus 0–1 and patients with > 2 versus ≤ 2 metastatic sites, whereas gender, age, clinical stage, and prior lines of chemotherapy were not associated with clinical outcome. The number of metastatic sites was a predictor of OS, consistent with the phase III study of apatinib [6]. Furthermore, the results of subgroup analyses according to baseline characteristics supported the interpretation of the lower OS observed in this study compared to the phase III study.

Apatinib was dosed at 850 mg once daily in the phase III study. Therefore, in the AHEAD study, apatinib was recommended at an initial dose of 850 mg, while the starting dose was decided by the investigator’s choice. A total of 98% of patients initiated apatinib at 500 mg, and subgroup analysis found the initial dose of apatinib did not affect OS or PFS. Notably, patients treated with an initial dose of ≤ 500 mg of apatinib had similar OS and PFS outcomes to those treated with an initial dose of 850 mg of apatinib in the phase III study [6]. In the phase III trial of apatinib in the treatment of advanced hepatocellular carcinoma (AHLEP) [21], the initial dose was recommended at 750 mg, while 45% of patients reduced apatinib to 500 mg due to adverse events. We analyzed the daily exposure of apatinib in the AHEAD study. The median daily exposure of apatinib was also 500 mg, without obvious implication on OS or PFS. The AHEAD study showed comparable toxicity results with the phase III study of apatinib [6]. These results suggested that a starting dose of 500 mg in clinical practice had an acceptable toxicity profile and had no impact on efficacy compared with 850 mg. A starting dose of 500 mg of apatinib is recommended in clinical settings for patients with gastric or gastroesophageal junction adenocarcinoma.

The strength of the AHEAD study was its large sample size and inclusion of patients excluded from phase III trials, providing a true presentation of the safety and efficacy of apatinib in treating advanced gastric cancer. Meanwhile, there were several limitations of this study. Firstly, AHEAD was a single-arm phase IV study lack of control group. Additionally, subgroup analyses were not prespecified and should be considered during the interpretation of the data.

## Conclusions

This large-scale, post-marketing phase IV study demonstrated the acceptable and manageable safety profile. It confirmed the efficacy of apatinib in treating advanced gastric or gastroesophageal junction adenocarcinoma after the failure of two or more lines of chemotherapy.

## Supplementary Information

**Additional file 1. Table S1.** Apatinib exposure; **Table S2.** Deaths thought to be related to apatinib; **Table S3.** Subgroup analyses of treatment-related adverse events; List 1. Institutions recruiting at least 20 patients.

**Additional file 2.** Protocol.

**Additional file 3.** AHEAD Study Raw Data.

## Data Availability

The study protocol is available as Supplementary Information in the Additional file 2. The data supporting this study’s findings are supplemented in Additional file 3.

## Abbreviations

ORR: Objective response rate
DCR: Disease control rate
PFS: Progression-free survival
OS: Overall survival
TRAEs: Treatment-related adverse events
VEGFR2: Vascular endothelial growth factor receptor 2
ECOG: Eastern Cooperative Oncology Group
PS: Performance status
MedDRA: Medical Dictionary for Regular Activities (MedDRA)
NCI-CTCAE: National Cancer Institute Common Terminology Criteria Adverse Events
RECIST: Response Evaluation Criteria in Solid Tumors
ITT: Intention-to-treat
TRSAEs: Treatment-related serious adverse events
