# Supplementary material for: Safety and efficacy of apatinib in patients with advanced gastric or gastroesophageal junction adenocarcinoma after the failure of two or more lines of chemotherapy (AHEAD): a prospective, single-arm, multicenter, phase IV study
Source: BMC Med. 2023 May 5;21:173. doi: 10.1186/s12916-023-02841-7 (PMC10163723; doi:10.1186/s12916-023-02841-7)
Supplement: Supplementary file 1 — Additional file 1. Table S1. Apatinib exposure; Table S2. Deaths thought to be related to apatinib; Table S3. Subgroup analyses of treatment-related adverse events; List 1. Institutions recruiting at least 20 patients. [file 12916_2023_2841_MOESM1_ESM.docx]

**Supplementary Information**

**Table S1. Apatinib exposure.**

|  | **Patients exposed to apatinib (n=1999)** |
| --- | --- |
| **Initial dose, *n* (%)** |  |
| 250 mg | 85 (4%) |
| 425 mg | 6 (0.3%) |
| 500 mg | 1795 (90%) |
| 675 mg | 7 (0.4%) |
| 750 mg | 51 (3%) |
| 850 mg | 55 (3%) |
| **Treatment duration, cycles** |  |
| Median (range) | 2 (1-37) |
| **Treatment duration, days** |  |
| Median (range) | 56 (1-1027) |
| **Daily exposure (mg)*** |  |
| Median (range) | 500 (36-850) |
| **Dose-intensity (%)** **†** |  |
| Median (range) | 92.6 (7.2-251.9) |
| **Dose interruption, *n* (%)** |  |
| Yes | 857 (43%) |
| No | 1042 (52%) |
| **Dose reduction, *n* (%)** |  |
| Yes | 422 (21%) |
| No | 1577 (79%) |

*Daily exposure was defined as the total dose received divided by the treatment duration.

**†**Dose-intensity was defined as the total dose received divided by the expected total dose.

**Table S2. Deaths thought to be related to apatinib (n=1999).**

|  | **Grade 5 events, *n* (%)** |
| --- | --- |
| **Patients with at least one event** | 57 (3%) |
| **Gastrointestinal disorders** | 24 (1%) |
| Gastrointestinal hemorrhage | 23 (1%) |
| Gastrointestinal perforation | 1 (<1%) |
| **General disorders and administration site conditions** | 10 (1%) |
| **Respiratory, thoracic, and mediastinal disorders** | 6 (<1%) |
| **Neoplasms benign, malignant and unspecified (including cysts and polyps)** | 6 (<1%) |
| **Nervous system disorders** | 5 (<1%) |
| Central nervous system bleeding | 2 (<1%) |
| **Hepatobiliary disorders** | 5 (<1%) |
| **Blood and lymphatic system disorders** | 5 (<1%) |
| **Metabolism and nutrition disorders** | 2 (<1%) |
| **Investigations** | 2 (<1%) |
| **Renal and urinary disorders** | 2 (<1%) |

**Table S3. Subgroup analyses of treatment-related adverse events.**

| **Subgroup, *n* (%)** | **Patients (n=1999)** | **Treatment-related adverse events**  **(any grade)** | **Treatment-**  **related adverse events**  **(grade ≥3)** | **Treatment-**  **related severe adverse events**  **(any grade)** |
| --- | --- | --- | --- | --- |
| **Gender** |  |  |  |  |
| Male | 1433 | 1265 (88%) | 741 (52%) | 193 (13%) |
| Female | 566 | 492 (87%) | 278 (49%) | 66 (12%) |
| **Age (years)** |  |  |  |  |
| ≤65 | 1413 | 1232 (87%) | 690 (49%) | 182 (13%) |
| >65 | 586 | 525 (90%) | 329 (56%) | 77 (13%) |
| **ECOG performance status** |  |  |  |  |
| 0-1 | 1684 | 1493 (89%) | 863 (51%) | 212 (13%) |
| 2-3 | 310 | 260 (84%) | 155 (50%) | 46 (15%) |
| **Tumour stage** |  |  |  |  |
| III | 70 | 55 (79%) | 34 (49%) | 13 (19%) |
| IV | 1927 | 1701 (88%) | 985 (51%) | 246 (13%) |
| **Number of metastatic sites** |  |  |  |  |
| ≤2 | 1290 | 1149 (89%) | 665 (52%) | 166 (13%) |
| >2 | 685 | 590 (86%) | 340 (50%) | 88 (13%) |
| **Prior lines of chemotherapy** |  |  |  |  |
| ≤2 | 1511 | 1329 (88%) | 765 (51%) | 205 (14%) |
| >2 | 486 | 428 (88%) | 254 (52%) | 54 (11%) |

ECOG, Eastern Cooperative Oncology Group.

**List 1. Institutions recruiting at least 20 patients.**

| **Institutions** | **Recruited patient number** |
| --- | --- |
| Shanxi Provincial Cancer Hospital | 137 |
| Anyang Cancer Hospital | 66 |
| The Affiliated Cancer Hospital of Zhengzhou University & Henan Cancer Hospital | 52 |
| Fudan University Shanghai Cancer Center | 44 |
| ZiBo Central Hospital | 39 |
| The Fourth Hospital of Hebei Medical University & Hebei Cancer Hospital | 38 |
| Xinxiang Central Hospital | 38 |
| The First Affiliated Hospital of USTC West District& Anhui Provicial Cancer Hospital | 34 |
| Tianjin Medical University Cancer Institute and Hospital, National Clinical Research Center for Cancer, Tianjin’s Clinical Research Center for Cancer, Key Laboratory of Cancer Prevention and Therapy | 33 |
| Zhejiang Cancer Hospital, Institute of Cancer and Basic Medicine, Chinese Academy of Sciences, Cancer Hospital of the University of Chinese Academy of Sciences | 33 |
| Cancer Center of Bayi Hospital, Nanjing Chinese Medicine University | 30 |
| Fujian Medical University Affiliated Union Hospital | 30 |
| Fujian Medical University Cancer Hospital, Fujian Cancer Hospital | 27 |
| Changzhi People's Hospital | 27 |
| Harbin Medical University Cancer Hospital | 26 |
| The Third Affiliated Hospital of Sun Yat-sen University | 26 |
| Heilongjiang Agricultural Reclamation Bureau General Hospital | 25 |
| Tangshan People's Hospital | 25 |
| Baotou Tumor Hospital | 25 |
| The First Affiliated Hospital of Xiamen University | 25 |
| Jiangyin People’s Hospital | 24 |
| The Affiliated Hospital of Guangdong Medical University | 24 |
| Xiangyang Central Hospital, Affiliated Hospital of Hubei University of Arts and Science | 24 |
| The Second People’s Hospital of Hefei | 23 |
| Weifang People’s Hospital | 22 |
| 105 Hospital of People's Liberation Army | 21 |
| The Second Affiliated Hospital Zhejiang University School of Medicine | 21 |
| The First Hospital of Jinlin Univercity | 20 |
| Shanxi Traditional Chinese Medical Hospital | 20 |
| Central Hospital Affiliated to Shandong First Medical University | 20 |
