# Supplementary material for: Safety and efficacy of apatinib in patients with advanced gastric or gastroesophageal junction adenocarcinoma after the failure of two or more lines of chemotherapy (AHEAD): a prospective, single-arm, multicenter, phase IV study
Source: BMC Med. 2023 May 5;21:173. doi: 10.1186/s12916-023-02841-7 (PMC10163723; doi:10.1186/s12916-023-02841-7)
Supplement: Supplementary file 2 — Additional file 2. Protocol. [file 12916_2023_2841_MOESM2_ESM.docx]

**A prospective, open-label, single-arm, multicenter, phase IV clinical study of apatinib mesylate tablets for the treatment of advanced gastric cancer or gastroesophageal junction adenocarcinoma following second-line chemotherapy failure in China**

**Study ID:** AHEAD

**Version Number/Date:** 2.2/12-21-2015

02-05-2015 version number 2.0

09-13-2015 version number 2.1

Sponsors: Hengrui Pharmaceuticals Co., Ltd.

Lead Sites: Fudan University Shanghai Cancer Center.

Cancer Center of Bayi Hospital Affiliated to Nanjing University of Chinese Medicine.

Statistical Agency: Department of Epidemiology and Biostatistics, Nanjing Medical University

**Disclosure Statement**

This study is sponsored by Hengrui Pharmaceuticals Co., Ltd.

Any and all information contained in this document shall be kept confidential and shall be regarded as the exclusive property of Hengrui Pharmaceuticals (or its affiliates). Such confidential information must be used only by intended recipients and shall not be disclosed, published, or otherwise transmitted to any unauthorized personnel in any form or for any reason without prior written permission of Hengrui Pharmaceuticals (or its affiliates). “Affiliates” include any groups, partnerships, or other entities that (i) directly or indirectly control Hengrui Pharmaceuticals or (ii) is directly or indirectly controlled by Hengrui Pharmaceuticals on or after the date of transmission. “Control” refers to direct or indirect ownership of 50% or more of total equity or direct or indirect voting rights in such groups, partnerships, or other entities.

**Table of Contents**

[**Protocol Signature Page** 6](#_Toc106010250)

[**Synopsis** 7](#_Toc106010251)

[**Study Schedule** 13](#_Toc106010252)

[**Abbreviations and Definitions** 14](#_Toc106010253)

[**1. Background and Principles** 17](#_Toc106010254)

[**1.1 Background** 17](#_Toc106010255)

[**1.1.1 Gastric cancer** 17](#_Toc106010256)

[**1.1.2 Treatments of gastric cancer** 18](#_Toc106010257)

[**1.1.3 Apatinib for the treatment of advanced gastric cancer** 18](#_Toc106010258)

[**1.2 Rationale** 22](#_Toc106010259)

[**1.3 Disease population** 23](#_Toc106010260)

[**2. Study Purpose** 23](#_Toc106010261)

[**2.1 Primary purpose** 23](#_Toc106010262)

[**2.2 Secondary purpose** 23](#_Toc106010263)

[**3. Study Design and Protocol** 23](#_Toc106010264)

[**3.1 Description of study design** 23](#_Toc106010265)

[**3.2 Dosing and drug management** 23](#_Toc106010266)

[**3.3 Study duration** 24](#_Toc106010267)

[**3.4 Study endpoints** 24](#_Toc106010268)

[**4. Study Population and Patient Selection** 25](#_Toc106010269)

[**4.1 Sample size** 25](#_Toc106010270)

[**4.2 Inclusion and exclusion criteria** 25](#_Toc106010271)

[**4.2.1 Inclusion criteria** 25](#_Toc106010272)

[**4.2.2 Exclusion criteria** 26](#_Toc106010273)

[**4.2.3 Drop-out/exclusion criteria** 26](#_Toc106010274)

[**4.2.4 Termination criteria:** 27](#_Toc106010275)

[**4.3 Enrollment** 27](#_Toc106010276)

[**5. Treatment Prescription** 27](#_Toc106010277)

[**6. Study Procedures and Data Collection** 27](#_Toc106010278)

[**6.1 Data collection plan** 27](#_Toc106010279)

[**6.2 Screening visit** 27](#_Toc106010280)

[**6.3 Visits during the treatment period (cycles 1-3)** 28](#_Toc106010281)

[**6.4 Additional visits** 28](#_Toc106010282)

[**6.5 Follow-up visits** 28](#_Toc106010283)

[**6.6 Follow-up of adverse events** 29](#_Toc106010284)

[**6.7 Procedures for patients who discontinue follow-up** 29](#_Toc106010285)

[**7. Data Management** 29](#_Toc106010286)

[**7.1 Data entry and modification** 29](#_Toc106010287)

[**7.2 Database security** 29](#_Toc106010288)

[**8. Safety Evaluation** 29](#_Toc106010289)

[**8.1 Monitoring adverse events** 29](#_Toc106010290)

[**8.2 Rating adverse events** 30](#_Toc106010291)

[**8.3 Recording adverse events** 30](#_Toc106010292)

[**8.4 Determining the relationship between adverse events and apatinib mesylate tablets** 30](#_Toc106010293)

[**8.5 Serious adverse events** 31](#_Toc106010294)

[**9. Statistical Processing** 33](#_Toc106010295)

[**9.1 Analysis populations** 33](#_Toc106010296)

[**9.2 Analysis variables** 33](#_Toc106010297)

[**9.2.1 Primary measures** 34](#_Toc106010298)

[**9.3 Statistical methods** 34](#_Toc106010299)

[**9.3.1 General principles:** 35](#_Toc106010300)

[**9.3.2 Primary analyses** 35](#_Toc106010301)

[**9.4 Determination of sample size** 36](#_Toc106010302)

[**10. Quality Control and Quality Assurance** 36](#_Toc106010303)

[**11. Ethics, Regulations, Administrative Principles** 36](#_Toc106010304)

[**11.1 Ethics principles** 36](#_Toc106010305)

[**11.2 Laws and regulations** 36](#_Toc106010306)

[**11.3 Data protection** 36](#_Toc106010307)

[**11.4 Confidentiality agreement** 36](#_Toc106010308)

[**11.5 Record maintenance** 37](#_Toc106010309)

[**11.6 Sponsor audit and regulatory inspection** 37](#_Toc106010310)

[**12. Protocol Amendment** 37](#_Toc106010311)

[**13. Use of Documents and Study Results** 37](#_Toc106010312)

[**13.1 Ownership and use of study data and study results** 37](#_Toc106010313)

[**13.2 Publications** 37](#_Toc106010314)

[**14. References** 39](#_Toc106010315)

**Protocol Signature Page**

I, a doctor/statistician involved in this study, have carefully read the study protocol.

I have discussed the study purpose and the protocol content in detail with the medical research director of the sponsors, Hengrui Pharmaceuticals Co., Ltd.

I agree to conduct this study according to the protocol and comply with its requirements, ethics regulations, and Good Clinical Practice (GCP). I also agree to keep the protocol content confidential and will not disclose it to any third party and acknowledge that the protocol content is used only in this study.

I understand that if the sponsor(s) decide to terminate or discontinue this study early at any time and for any reason, they must notify me in advance in writing. Similarly, if I decide to withdraw from this study, I will also promptly notify Hengrui Pharmaceuticals Co., Ltd. in writing.

**Data Privacy Policy:**

I have been informed that the sponsors will collect and use nonconfidential personal and professional information provided by me and disclose it to relevant internal trial management personnel. The above information will be recorded in a database and will be used only for the sole purpose of site initiation and study conduct.

Institution: ________________

Signature:_______________

Date:_______________

**Synopsis**

| Study Title | | A prospective, open-label, single-arm, multicenter, phase IV clinical study of apatinib mesylate tablets for the treatment of advanced gastric cancer or gastroesophageal junction adenocarcinoma following second-line chemotherapy failure in China |
| --- | --- | --- |
| Protocol Number | | AHEAD |
| Version Number/Date | | 2.2/12-21-2015 |
| Sponsor(s) | | Hengrui Pharmaceuticals Co., Ltd. |
| Nature | | Postmarketing phase IV clinical study of a new drug in accordance with the requirements for approval by the National Medical Products Administration |
| Subjects | | Patients with advanced gastric cancer or gastroesophageal junction adenocarcinoma following at two lines chemotherapy failure |
| Study  Objectives | Primary Objective | To evaluate the safety of apatinib mesylate tablets in patients with advanced gastric cancer or gastroesophageal junction adenocarcinoma following at least two lines chemotherapy failure |
|  | Secondary Objective | To evaluate the effectiveness of apatinib mesylate tablets in patients with advanced gastric cancer or gastroesophageal junction adenocarcinoma following second-line chemotherapy failure |
| Study Design | | Prospective, open-label, single-arm, multicenter phase IV clinical study in China |
| Planned Enrollment | | 2000 patients |
| Number of Sites | | 100 |
| Principal Investigator(s) | | Prof. Jin Li; Prof. Shukui Qin |
| Lead Site(s) | | Fudan University Shanghai Cancer Center; PLA 81 Hospital |
| Inclusion and Exclusion Criteria | | **Inclusion Criteria:**  1. Age: 18 to 75 years old, men and women;  2. Pathologically and/or cytologically confirmed advanced gastric cancer or gastroesophageal junction adenocarcinoma, with an extragastric measurable lesion (spiral computed tomography (CT) ≥ 10 mm, conforming to Response Evaluation Criteria in Solid Tumors version 1.1 (RECIST 1.1));  Note: Patients without any gastric or extragastric measurable lesion but with an observable lesion may be enrolled after a comprehensive evaluation by investigators from each institution to observe quality of life (QoL) and survival. These patients will be excluded from the objective response evaluation.  3. Patients with advanced gastric cancer or gastroesophageal junction adenocarcinoma following ≥ second-line chemotherapy failure;  Note: (1) Definition of treatment failure: disease progression during treatment or tumor relapse and metastasis after treatment, or intolerable toxicity; (2) for progressive disease, each line of treatment is ≥ 1 cycle or longer with one or more chemotherapy drugs; pilot adjuvant/neoadjuvant therapy is allowed and will be considered first-line systemic chemotherapy for progressive disease in the case of tumor relapse and metastasis during treatment or within 24 weeks after treatment; and (3) upfront therapy can be chemotherapy combined with molecular targeted drugs.  4. Eastern Cooperative Oncology Group (ECOG) performance status (PS) score: 0-2;  5. Normal vital organ functions based on the following standards:  (1) Complete blood count (no blood transfusion within 14 days):  a. Hemoglobin (HB) ≥ 90 g/L;  b. Absolute neutrophil count (ANC) ≥ 1.5 × 10^9^/L; and  c. Platelet (PLT) count ≥ 80 × 10^9^/L.  (2) Serum biochemistry:  a. Bilirubin (BIL) < 1.5x the upper limit of normal (ULN);  b. Alanine aminotransferase (ALT) and aspartate aminotransferase (AST) < 2.5 × ULN; or < 5 × ULN in the case of liver metastasis; and  c. Serum creatinine (Cr) ≤ 1 × ULN, endogenous creatinine clearance ≥ 50 ml/min (Cockcroft-Gault formula).  6. Expected survival ≥ 3 months;  7. The treating physician planned to use apatinib mesylate tablets for treatment;  8. Voluntary participation, with a signed informed consent form (ICF); and  9. Women with childbearing potential must have a negative pregnancy test (serum or urine) within 7 days of enrollment and be willing to use appropriate birth control throughout the observation period and for 8 weeks after the last dose of apatinib mesylate tablets. Men must be surgically sterile or agree to use appropriate birth control throughout the observation period and for 8 weeks after the last dose of apatinib mesylate tablets.  **Exclusion criteria:**  1. Uncontrolled hypertension despite treatment with antihypertensive drugs (systolic blood pressure > 140 mmHg/diastolic blood pressure > 90 mmHg), grade ≥ Ⅱ coronary heart disease, arrhythmia (including the corrected QT (QTc) prolongation > 450 ms in men or > 470 ms in women), or cardiac insufficiency;  2. Presence of several factors that may affect the absorption of oral drugs (such as swallow difficulty, nausea and vomiting, chronic diarrhea, and intestinal obstruction);  3. Important: patients at risk of gastrointestinal bleeding may not participate, including those with (1) active digestive ulcers, with fecal occult blood (++); (2) a history of melena and hematemesis within 3 months; (3) for patients with fecal occult blood (+) and an unresected primary gastric tumor, if endoscopy indicates ulcerative gastric cancer with the possibility of lesion bleeding in the opinion of the treating physician;  4. Coagulopathy (international normalized ratio (INR) > 1.5 × ULN, activated partial thromboplastin time (APTT) > 1.5 × ULN), with bleeding tendency;  5. Symptomatic central nervous system metastasis;  6. Pregnant or nursing women; and  7. Any patient deemed unsuitable for this study by the treating physician.  **Drop-out/exclusion criteria:**  1. Failure to take the drug per protocol, resulting in exclusion from effectiveness and/or safety evaluation;  2. Major protocol deviation: use of chemotherapy drugs and/or herbal preparations indicated for gastric cancer while in the study.    **Termination criteria:**  1. Subject withdraws informed consent and requests an early exit;  2. Medical imaging indicates disease progression;  3. Becoming pregnant during the study;  4. Intolerable toxicity after two dose adjustments; and  5. In the opinion of the investigator, other circumstances that necessitate study exit. |
| Dosing Schedule | | Apatinib mesylate tablets: 850 mg, po, qd  28-day observation cycles.  Note: To ensure patient safety and improve compliance, for female patients at an advanced age, with poor conditions or a small stature (≤ 50 kg) and who have unresected primary gastric cancer after four lines of chemotherapy, the initial dose may be reduced to 500 mg po qd, which may be increased as appropriate after 1-2 weeks or remain unchanged. The actual dose is recorded. |
| Study Endpoints | | **Primary endpoint:**  To observe and evaluate the safety of apatinib mesylate tablets in patients with advanced gastric cancer or gastroesophageal junction adenocarcinoma, including the incidence, severity, correlation, risk factors, measures taken, and outcome of adverse events (AEs);  Special attention should be paid to specific AEs, including hypertension, bleeding, proteinuria, and hand-foot syndrome.  **Secondary endpoint:**  To observe and evaluate the effectiveness of apatinib mesylate tablets for the treatment of advanced gastric cancer or gastroesophageal junction adenocarcinoma, including objective response rate (ORR), disease control rate (DCR), progression-free survival (PFS), overall survival (OS), and QoL.  Note: It is currently believed that RECIST criteria are used mainly for the observation and evaluation of objective responses of cytotoxic chemotherapy drugs and cannot comprehensively or accurately reflect the efficacy of molecular targeted drugs. Apatinib mesylate tablets may be continued, under close monitoring, if medical imaging indicates significant degeneration, necrosis, or liquefaction in the lesion even though the lesion has grown or if clinical symptoms and/or QoL are improved, the treating physician believes that treatment continuation will bring survival benefits, and the patient has no significant adverse reactions and is willing to continue the treatment. |
| Safety Measures | | Vital signs and laboratory tests to determine the correlation between AEs and apatinib mesylate tablets and the correlation between and serious adverse events (SAEs) and apatinib mesylate tablets. |
| Study Duration | | The entire duration is expected to be 36 months. Each patient will be followed up until death or study withdrawal or 6 months after the last eligible patient is enrolled.  Planned first-patient-in/start date: April 1, 2015  Planned last-patient-in date: April 1, 2018  Planned last-patient-out/end date: October 1, 2018 (6 months after the last eligible patient is enrolled)  Planned database lock: December 31, 2018  Planned reporting date: April 1, 2019 |
| Statistical Processing | | **Main analyses:**  The safety analysis uses mainly descriptive statistics, including tabulation of the incidence, severity, correlation, risk factors, measures taken, and outcomes of AEs observed in this study.  **Secondary analyses:**  RR and DCR and their 95% confidence intervals (CIs) will be calculated; the Kaplan-Meier method will be used to plot OS curves and PFS curves, and median overall survival (mOS) and median progression-free survival (mPFS) and their 95% CIs will be estimated. |

**Study Schedule**

| **Item** | **Within 2 weeks before treatment** | **Within 1 week before treatment** | **Treatment Cycle** | | | | | | | | | | | |
| --- | --- | --- | --- | --- | --- | --- | --- | --- | --- | --- | --- | --- | --- | --- |
|  |  |  | **Cycle 1** | | | | **Cycle 2** | **Cycle 3** | **Cycle 4** | | | | **Cycle 5** | **Cycle 6+** |
|  |  |  | 1 w | 2 w | 3 w | 4 w |  |  | 1 w | 2 w | 3 w | 4 w |  |  |
| Signed informed consent form | **Ⅹ** |  |  |  |  |  |  |  |  |  |  |  |  |  |
| History of previous treatments |  | **Ⅹ** |  |  |  |  |  |  |  |  |  |  |  |  |
| Vital signs |  | **Ⅹ** |  |  |  | **Ⅹ** | * | * |  |  |  | **Ⅹ** | # | # |
| Physical examination |  | **Ⅹ** |  |  |  | **Ⅹ** | * | * |  |  |  | **Ⅹ** | # | # |
| Chest X-ray |  | **Ⅹ f** |  |  |  |  |  |  |  |  |  |  |  |  |
| Blood pressure **a** |  | **Ⅹ** | **3** | **3** | **2** | **2** | * | * | **1** | **1** | **1** | **1** | # | # |
| Echocardiography |  | **Ⅹ** |  |  |  | **Ⅹ b** | * | * |  |  |  | **Ⅹ b** | # | # |
| Coagulation |  | **Ⅹ** |  |  |  | **Ⅹ** | * | * |  |  |  | **Ⅹ** | # | # |
| Electrocardiogram (ECG) | **Ⅹ** |  |  |  |  | **Ⅹ** | * | * |  |  |  | **Ⅹ** | # | # |
| ECOG PS score |  | **Ⅹ** |  |  |  | **Ⅹ** | * | * |  |  |  | **Ⅹ** | # | # |
| Complete blood count |  | **Ⅹ** |  | **Ⅹ** |  | **Ⅹ** | * | * |  |  |  | **Ⅹ** | # | # |
| Urinalysis |  | **Ⅹ** |  | **Ⅹ** |  | **Ⅹ** | * | * |  |  |  | **Ⅹ** | # | # |
| Stool test |  | **Ⅹ** |  | **Ⅹ** |  | **Ⅹ** | * | * |  |  |  | **Ⅹ** | # | # |
| Liver and kidney function, electrolytes |  | **Ⅹ** |  |  |  | **Ⅹ** | * | * |  |  |  | **Ⅹ** | # | # |
| Tumor marker (CEA) |  | **Ⅹ** |  |  |  |  | **Ⅹ** | **Ⅹ** |  |  |  |  | **Ⅹ** | **&** |
| Imaging  (X-ray/CT/MRI/ultrasound B) | **Ⅹ c** |  |  |  |  |  | **Ⅹ** | **Ⅹ d** |  |  |  |  | **Ⅹ** | **&** |
| Gastroscopy |  | **Ⅹ e** |  |  |  |  |  |  |  |  |  |  |  |  |
| Quality of life score |  | **Ⅹ** |  |  |  |  | **Ⅹ** | **Ⅹ** |  |  |  |  | **Ⅹ** | **&** |
| Adverse events |  |  | **Ⅹ** | **Ⅹ** | **Ⅹ** | **Ⅹ** | * | * | * | * | * | * | * | * |
| Concomitant medications |  |  | **Ⅹ** | **Ⅹ** | **Ⅹ** | **Ⅹ** | * | * | * | * | * | * | * | * |

Note: **All measures and exams/tests, including imaging studies, should be performed according to the study schedule for each cycle and will not be affected by drug withdrawal.**

(1) *: same as cycle 1; (2) #: same as cycle 4; (3) &: every 2 cycles; (4) a: 3: three times, 2: twice, 1: once; (5) echocardiography b: used in the case of abnormal ECG; (6) c: including chest, abdomen, and pelvic computed tomography (CT) or magnetic resonance imaging (MRI); (7) d: X-ray or ultrasound B during the study if chest, abdomen, or pelvic imaging is negative at screening. In the case of positive X-ray or ultrasound B, CT or MRI should be performed to confirm; (8) e: gastroscopy is required for patients with fecal occult blood (+) and an unresected primary gastric tumor; (9) f: perform as needed.

**Abbreviations and Definitions**

The following abbreviations and specific terms are used in this protocol.

| **Abbreviations and Specific Terms** | **Definition** |
| --- | --- |
| AE | Adverse event |
| AGC | Advanced gastric cancer |
| AKP | Alkaline phosphatase |
| ALB | Albumin |
| ALT | Alanine aminotransferase |
| ANC | Absolute neutrophil count |
| APTT | Activated partial thromboplastin time |
| AST | Aspartate aminotransferase |
| BIL | Bilirubin |
| BUN | Blood urea nitrogen |
| Ca | Calcium |
| CEA | Carcinoembryonic antigen |
| CI | Confidence interval |
| Cl | Chlorine |
| Cr | Creatinine |
| CR | Complete response |
| CRF | Case report form |
| CT | Computed tomography |
| DBIL | Direct bilirubin |
| DCR | Disease control rate |
| DRQ | Data-response questions |
| ECOG PS | Eastern Cooperative Oncology Group Performance Status |
| FAS | Full analysis set |
| Fbg  GC | Fibrinogen  Gastric cancer |
| GCP | Good Clinical Practice |
| Hb | Hemoglobin |
| HFS | Hand-foot syndrome |
| HP | *Helicobacter pylori* |
| ICF | Informed consent form |
| INR | International normalized ratio |
| IRB | Institutional Review Board |
| LDH | Lactate dehydrogenase |
| LVEF | Left ventricular ejection fraction |
| K | Potassium |
| mPFS | Median progression-free survival |
| MRI | Magnetic resonance imaging |
| Na | Sodium |
| NMPA | National Medical Products Administration |
| NCI-CTC | National Cancer Institute-Common Toxicity Criteria |
| ORR | Objective response rate |
| OS | Overall survival |
| P | Phosphorus |
| PD | Progressive disease |
| PDGFR | Platelet-derived growth factor receptor |
| PFS | Progression-free survival |
| PLT | Platelet count |
| PR | Partial response |
| PT | Prothrombin time |
| qd | Once a day |
| QoL | Quality of life |
| RBC | Red blood cell |
| RECIST | Response Evaluation Criteria in Solid Tumors |
| SAE | Serious adverse event |
| SD | Stable disease |
| SOC | System organ classification |
| TBIL | Total bilirubin |
| TP | Total protein |
| TT | Thrombin time |
| ULN | Upper limit of normal |
| VEGFR | Vascular endothelial growth factor receptor |
| WBC | White blood cell |
| γ-GT | Gamma-glutamyltransferase |
|  |  |

**1. Background and Principles**

**1.1 Background**

**1.1.1** **Gastric cancer**

Gastric cancer (GC) is one of the most common gastrointestinal malignancies. It originates from mucosal epithelial cells at the surface of the gastric wall and can occur in any part of the stomach (e.g., the pylorus area of the gastric antrum, the fundus cardia and the gastric body). GC can invade different depths and breadths of the gastric wall, and adenocarcinoma accounts for 95% of all GC. Focal mucosal or submucosal lesions are classified as early GC, while muscular or extragastric lesions are considered advanced gastric cancer (AGC). Gross or gastroscopic observation shows many forms of GC, such as superficial, mass, ulcerative, infiltrating, and ulcer cancer (chronic gastric ulcer canceration) ^[1]^.

According to the World Health Organization (WHO), worldwide, approximately 989600 individuals are diagnosed with GC each year, making GC the fourth leading malignancy ^[2,3]^. Moreover, 738000 individuals die from GC each year, making it the second leading cancer of cancer mortality ^[4]^. Therefore, GC poses a serious threat to human health and life.

The prevalence of GC is high in East Asia, South America, and Eastern Europe. Recent statistics show that more than 400000 individuals are diagnosed with GC each year in China, with a prevalence of 36.21 per 100000 individuals (the second most common cancer), accounting for nearly 50% of all GC cases worldwide. Moreover, approximately 350000 individuals die of GC each year in China, i.e., a mortality rate of 25.88 per 100000 individuals (the third highest mortality rate for cancer). The WHO World Cancer Report (2014) indicated that in 2012, more than 40% of new GC cases and deaths occurred in China and that GC has become one of the three most common cancers in China.

According to the *Chinese Cancer Registry Annual Report*, GC has the following epidemiologic features in China: 1) widespread geographic distribution, especially in the northwest and the southeast coastal areas, with scattered high-prevalence areas and significant regional variations; 2) 60% to 70% higher prevalence in rural areas than in urban areas and occurrence in any age group, especially those aged 40 to 60 years. Mortality increases with age, and the prevalence and mortality are twice as high for men than for women ^[5]^; 3) the mortality rate is 40.8 per 100000 individuals for men and 18.6 per 100000 individuals for women, which is approximately 4 to 8 times higher than that in Europe and North America.

GC may be related to many factors, including lifestyle, diet, environmental factors, genetic susceptibility, and mental factors as well as chronic gastritis, gastric polyps, gastric mucosal dysplasia and intestinal metaplasia, postoperative gastric stumps, and long-term *Helicobacter pylori* (HP) infection ^[6]^.

**1.1.2 Treatments of gastric cancer**

For patients with AGC, the median overall survival (mOS) is only 3 to 4 months without chemotherapy. Numerous clinical studies show that chemotherapy has a certain palliative effect on AGC and extends mOS to 6 to 10 months ^[7]^.

Chemotherapy drugs with proven effects on AGC include fluorouracil-based drugs, platinum-based drugs, anthracyclines, and camptothecins, as well as mitomycin and etoposide. First-line regimens are usually fluorouracil- and/or platinum-based combination therapies; second-line treatment usually uses drugs (monotherapy or combination therapy) not used during first-line treatment, such as taxanes and irinotecan, but no standard of care has been established ^[8,9]^. In the case of second-line chemotherapy failure, many patients with significant symptoms are still in good physical condition and expect to live and receive effective treatments; therefore, it is urgent to establish new treatments/regimens to benefit these patients.

With advancements in and a deeper understanding of molecular biology and molecular pathology in tumor occurrence, development, and metastasis, molecular targeted therapy has become a novel treatment in addition to surgery, chemotherapy, and radiotherapy. Targeted therapy was once considered promising for GC, but a series of studies showed that bevacizumab ^[10]^, sunitinib ^[11-13]^, and sorafenib ^[14-15]^ were ineffective for AGC. Trastuzumab-based combination chemotherapy is proven to be effective in 10% to 15% of GC patients with HER-2 overexpression and has been approved in several countries.

**1.1.3 Apatinib for the treatment of advanced gastric cancer**

Apatinib mesylate is a small-molecule vascular endothelial growth factor receptor (VEGFR) tyrosine kinase inhibitor that has been independently developed by Hengrui Pharmaceuticals Co., Ltd. It is a new derivative of vatalanib **(**PTK787). The chemical name is methanesulfonic acid N-[4-(cyanocyclopentyl) phenyl][2-[(4-pyridylmethyl)amino](3-pyridine)]carboxamide, the molecular formula is C_25_H_27_N_5_O_3_S, and the molecular weight is 493.58 (methanesulfonate).

Apatinib effectively inhibits VEGFR at a low concentration; at higher concentrations, it also inhibits platelet-derived growth factor receptor (PDGFR) and c-Kit and c-Src kinases. Activity testing shows that its binding affinity to VEGFR is more than 10 times stronger than that of PTK787. Apatinib acts on the intracellular adenosine triphosphate-binding site of the tyrosine receptor. Pharmacodynamic studies show that apatinib inhibits the activity of VEGFR tyrosine kinase, blocks signal transduction after VEGF binding, and strongly inhibits tumor angiogenesis. Preclinical studies show that apatinib is significantly more effective than PTK787 for treating nude mice xenografts of human GC, colon cancer, and lung cancer, with an inhibition rate of up to 80.7% for GC.

**1.1.3.1 Apatinib phase I clinical study**

A phase Ⅰ pharmacokinetic study in healthy subjects indicated that after a single oral dose of apatinib on an empty stomach, the prodrug was rapidly absorbed, its plasma concentration peaked after an average of 1.7 to 2.3 hours, and the mean elimination half-life was 7.9 to 9.4 hours. The plasma concentrations of the four metabolites peaked after an average of 2.7 to 5.8 hours, and the elimination half-life was 9 to 18 hours. No significant sex variation was observed in pharmacokinetic parameters in healthy subjects. The plasma exposure of the prodrug and the four metabolites was proportional to the dose in the 250 mg and 500 mg dose groups, with no further increase in the 750 mg dose group. In human metabolism and elimination studies, UPLC/Q-TOF MS was used after the oral administration of apatinib to detect the prodrug and its main metabolites. The results showed that 77% of the total dose was eliminated via urine and feces, of which, 69.8% was eliminated via feces, indicating that apatinib is mainly excreted in feces after oral administration.

An apatinib phase Ⅰa clinical study enrolled 19 patients with advanced cancer. Apatinib was well tolerated after one cycle (4 weeks), and most adverse events (AEs) were mild to moderate. Drug-related adverse reactions mainly included hand-foot syndrome (HFS), hypertension, leukopenia, oral mucositis, thrombocytopenia, fatigue, elevated bilirubin, headache, low back pain, nausea, tongue pain, precordial pain, chest pain, elevated liver enzymes, upper abdominal discomfort, hoarseness, stomach upset, proteinuria, and sinus bradycardia. In this study, preliminary objective response evaluations showed that among the three patients who received the clinical recommended dose of 750 mg/day, one patient achieved a partial response (PR), one had stable disease (SD), and one had progressive disease (PD). Among the six patients who received the maximum tolerated dose of 850 mg/day, two patients achieved a PR, three had SD, and one had PD. Pharmacokinetic studies of apatinib showed that after single-dose oral administration (500, 750, 850 mg/day), the mean plasma concentration of and area under the drug-time curve for apatinib and M1 (a major metabolite of YN968D1 in the body) increased linearly with the dose in both men and women. Apatinib exposure varies somewhat between men and women. Specifically, sex variation was not observed in the 500 and 750 mg/day dose groups but was observed in the 850 mg/day group (the level of exposure was significantly higher in women than in men). Moreover, in the 850 mg/day group, apatinib exposure increased in a nonlinear manner in women, and eating and sequence of medications had no significant effect on YN968D1 and M1 exposure in the body (*P* > 0.05).

**1.1.3.2 Phase IIa/IIb study of apatinib for the treatment of advanced gastric cancer**

The phase Ib/IIa clinical study was a single-center study conducted at the Drug Clinical Trial Center, Fudan University Shanghai Cancer Center. A total of 65 patients were enrolled. The complete response (CR) rate was 1.54%, the PR rate was 12.31%, the SD rate was 66.15%, and the disease control rate (DCR) was up to 80%.

Subsequently, as principal investigators (PIs), Prof. Lin Li of the Department of Oncology, Fudan University Shanghai Cancer Center (Shanghai, China), and Prof. Shukui Qin of the Cancer Center, PLA 81 Hospital (Nanjing, China), conducted a multicenter, randomized, double-blind, parallel-controlled, phase IIb clinical study in China to investigate the effect of apatinib in patients with AGC following failure of standard chemotherapy (second-line chemotherapy with taxanes, platinum-based drugs, and fluorouracils). A total of 141 patients were enrolled and randomized into one of the three groups: placebo control group, apatinib 850 mg qd group, and apatinib 425 mg bid group. The results demonstrated a superior objective response rate (ORR) (6.38% for 850 mg qd, 13% for 425 mg bid) and survival benefits for apatinib. The mOS was 2.50, 4.83, and 4.27 months, respectively; and the median progression-free survival (mPFS) was 1.40, 3.67, and 3.20 months. These data indicate that apatinib tablets (850 mg qd and 425 mg bid) were more effective than placebo; 425 mg bid was superior to 850 mg qd with respect to ORR but inferior with respect to progression-free survival (PFS), overall survival (OS), and DCR, suggesting that a higher ORR does not necessarily translate into a longer survival. Placebo and apatinib 850 mg qd were safer than apatinib 425 mg bid. Pharmacokinetic studies showed that the Cmin, Cav, and AUC0-24 of the prodrug and metabolite M1 were higher in the 425 mg bid group than in the 850 mg qd group. Moreover, 850 mg qd was more effective than 425 mg bid in inhibiting serum VEGFR-2 expression, and both dosing regimens upregulated serum VEGF expression. Based on these data, apatinib 850 mg po qd was used in the phase Ⅲ clinical study.

**1.1.3.3 Phase III study of apatinib for the treatment of advanced gastric cancer**

Based on the results of the above phase I and II clinical studies, Prof. Jin Li and Prof. Shukui Qin conducted a multicenter, randomized, double-blind, parallel-controlled, superiority phase III clinical study in China (ClinicalTrials.gov registration number: NCT01512745) to further evaluate and validate the efficacy and safety of apatinib mesylate tablets versus placebo in patients with AGC. The primary endpoint was OS; the secondary endpoints were PFS, ORR, DCR, quality of life (QoL), and safety.

The phase III study enrolled a total of 273 AGC patients who did not respond to second-line systemic chemotherapy. The patients were centrally randomized at 2:1 into an experimental group (n = 181; apatinib) or placebo control group (n = 92; a placebo, namely, an apatinib simulation agent). The dose was 850 mg po, qd; each cycle was 28 days. Objective response was evaluated in accordance with RECIST 1.1, and AEs were closely monitored and evaluated in accordance with the National Cancer Institute (NCI) Common Terminology Criteria for Adverse Events, version 3.0 (CTCAE 3.0). The primary endpoint was OS; the secondary endpoints were PFS, DCR, ORR, QoL, and safety.

**Results:** After randomization, six untreated patients were excluded from analyses. A total of 267 randomized patients received at least one dose, including 176 patients in the experimental group and 91 patients in the placebo control group. Baseline data were comparable between the two groups. As of May 23, 2013, full analysis set (FAS) analysis, without considering crossover, showed that the mOS was 6.5 months in the experimental group and 4.7 months in the control groups, indicating that apatinib significantly extended survival by 1.8 months (*P* = 0.0149). Per-protocol set analysis indicated that the mOS was 7.6 for the experimental group and 5.0 months for the placebo control group, indicating that apatinib significantly extended survival by 2.6 months (*P* = 0.0027). When considering crossover, the mOS was 4.5 months in the control group after the data were adjusted for subjects who crossed over to the experimental group and was 2.0 months longer in the experimental group (HR = 0.646, *P* = 0.006), indicating that apatinib extended the mOS in the experimental group. The mPFS was 2.6 months in the experimental group and 1.8 months in the control group (*P* < 0.0001); the ORR was 2.84% in the experimental group and 0.00% in the control group (*P* = 0.1695); and the DCR was 42.05% in the experimental group and 8.79% in the control group (*P* < 0.0001). No significant between-group difference was observed in the change in QoL score (*P* > 0.05). For safety, apatinib was well tolerated in the experimental group, and the type and incidence of AEs were consistent with the findings in the apatinib phase II clinical study and with those for similar drugs on the market. Common AEs were leukopenia, neutropenia, thrombocytopenia, proteinuria, hypertension, HFS, fatigue, loss of appetite, diarrhea, and hoarseness; no unexpected AEs were observed. The incidence rate of AEs was 98.30% in the experimental group and 90.11% in the control group (*P* = 0.0038); the incidence of grade 3/4 AEs was 60.23% in the experimental group and 41.76% in the control group (*P* = 0.0000); the incidence of serious adverse events (SAEs) was 15.34% in the experimental group and 16.48% in the control group (*P* = 0.8598); and the incidence of drug-related SAEs was 6.25% in the experimental group and 6.59% in the control group (*P* = 1.0000). Most AEs were expected, tolerable, reversible, and controllable.

**Conclusion:** Apatinib exhibited a certain objective response and significant survival benefits in patients with AGC. Both primary and secondary efficacy measures, including OS, PFS, ORR, and DCR, were superior in the apatinib treatment group, indicating definitive survival benefits of apatinib. Moreover, apatinib was safe and well tolerated. The type and incidence of apatinib-related AEs were consistent with the adverse reactions of similar small-molecule VEGFR inhibitors on the market and the results of the apatinib phase II clinical study. No unexpected specific AEs were observed, and most AEs were reversed or controlled after drug discontinuation, dose reduction, or symptomatic care. Apatinib had no adverse effect on QoL. Therefore, apatinib can be used in clinical practice at the recommended dose of 850 mg qd ^[16.17]^. At the American Society of Clinical Oncology (ASCO) annual conference (June 2014), Prof, Jin Li of Fudan University Shanghai Cancer Center, on behalf of the study team, presented the results of the phase III clinical study of apatinib for the treatment of AGC, which attracted a great deal of attention ^[16]^.

Based on the above studies, the National Medical Products Administration (NMPA) approved apatinib tablets on October 17, 2014, as a category 1.1 new drug for the treatment of AGC that progresses after second-line chemotherapy failure.

**1.2 Rationale**

GC is a common malignancy worldwide. In China, its prevalence is especially high, and it is difficult to treat. Before apatinib was approved, no effective third-line treatment was available for AGC following second-line chemotherapy failure. During the same period of the clinical study of apatinib for the treatment of AGC, in 2014, a clinical study of ramucirumab (as monotherapy), a VEGFR-2 monoclonal antibody developed by Eli Lilly and Company (USA), was successful in patients with AGC or gastroesophageal junction cancer who did not respond to previous fluorouracil- or platinum-based chemotherapy (REGARD) ^[18]^, paving the way for ramucirumab to become the second (after trastuzumab) US FDA-approved targeted drug for GC and highlighting the importance of antitumor angiogenesis drugs for AGC, especially the feasibility of VEGFR-2 as a treatment target. However, ramucirumab is ineffective in Asian patients and is not yet available in China. Apatinib has been successful developed and has been approved by the NMPA; however, premarketing clinical studies were efficacy studies in certain patient populations with many exclusion criteria and may not reflect real-world data. Therefore, it is important to conduct prospective postmarketing studies to fill the gap between clinical practice and premarketing clinical studies to ensure rational drug use, safety, and effectiveness. During apatinib market authorization, the NMPA required Hengrui Pharmaceuticals Co., Ltd. To develop a rigorous risk management plan, conduct a phase IV clinical study to further observe and evaluate the large-scale postmarketing application of apatinib, collect detailed safety and efficacy data, and submit reports to the NMPA in a timely manner (see Annex).

**1.3 Disease population**

Patients with AGC or gastroesophageal junction adenocarcinoma following second-line chemotherapy failure

**2. Study Purpose**

**2.1 Primary purpose**

To evaluate the safety of apatinib mesylate tablets for the treatment of AGC or gastroesophageal junction adenocarcinoma following second-line chemotherapy failure.

**2.2 Secondary purpose**

To evaluate the effectiveness (OS, PFS, ORR, DCR, and QoL) of apatinib mesylate tablets for the treatment of AGC or gastroesophageal junction adenocarcinoma following second-line chemotherapy failure

**3. Study Design and Protocol**

**3.1 Description of study design**

This is a multicenter, prospective, open-label, single-arm, phase Ⅳ clinical study in China that is designed to evaluate the safety and effectiveness of apatinib mesylate tablets for the treatment of AGC or gastroesophageal junction adenocarcinoma following second-line chemotherapy failure. Therefore, the data collected and reported will comprehensively reflect the real-world data of apatinib mesylate tablets for the treatment of AGC or gastroesophageal junction adenocarcinoma following second-line chemotherapy failure.

**3.2 Dosing and drug management**

**3.2.1 Dosing schedule**

Apatinib mesylate tablets, 850 mg po qd, should be taken with warm boiled water 30 minutes after eating. The drug should be taken at the same time on each day whenever possible.

Note: To ensure patient safety and improve compliance, for female patients at an advanced age, with a poor condition or a small stature (≤ 50 kg) and who have unresected primary GC after four lines of chemotherapy, the initial dose may be reduced to 500 mg po qd, which can be increased as appropriate after 1-2 weeks or remain unchanged. The actual dose should be recorded.

**3.2.2 Dosing cycle**

Each dosing and observation cycle is 28 days. Responders (CR, PR, and SD) continue to take the drug until PD or death; the drug will be discontinued in the case of intolerable toxicity, patient withdrawal, or PD.

For patients with radiographically confirmed PD, the PI in the institution will promptly review the images and patient condition. Patients can continue treatment under close monitoring and follow-up, if they do not have intolerable reactions, their symptoms or QoL improved, the treating physician (investigator) believes that continuing to receive treatment increase survival benefits, and both the patient and PI agree to continue the treatment.

**3.2.3 Dose adjustment and drug discontinuation**

Refer to the NMPA-approved apatinib mesylate tablets package insert (see Annex).

**3.2.4 Drug management**

Apatinib mesylate tablets should be stored at room temperature (25°C or below), away from light in a sealed container. The shelf life is 2 years (tentative).

**3.3 Study duration**

The study is expected to last 5 years, including a 36-month enrollment period. After the end of treatment, each patient will be followed up until death or study withdrawal or 6 months after the last eligible patient is enrolled.

⚫ Planned first-patient-in/start date: April 1, 2015

⚫ Planned last-patient-in date: April 1, 2018

⚫ Planned last-patient-out/end date: October 1, 2018 (6 months after the last eligible patient is enrolled)

⚫ Planned database lock: December 31, 2018

⚫ Planned reporting date: April 1, 2019

**3.4 Study endpoints**

**Primary endpoint:**

⚫ **Safety:**

The incidence, severity, correlation, risk factors, measures taken, and outcomes of all AEs will be closely monitored and recorded in accordance with NCI CTCAE 4.0. Special attention will be paid to specific AEs, such as hypertension, bleeding, proteinuria, and HFS.

**Secondary endpoints:**

⚫ **Effectiveness:**

OS and PFS; ORR and DCR in accordance with RECIST 1.1; and QoL score

**4. Study Population and Patient Selection**

**4.1 Sample size**

The study will be conducted with 2000 patients at 100 sites across China.

**4.2 Inclusion and exclusion criteria**

**4.2.1 Inclusion criteria**

1. Age: 18 to 75 years old, men or women;

2. Pathologically and/or cytologically confirmed advanced gastric or gastroesophageal junction adenocarcinoma, with an extragastric measurable lesion (spiral computed tomography (CT) ≥ 10 mm, conforming to RECIST 1.1);

Note: Patients without any gastric or extragastric measurable lesion but with an observable lesion can be enrolled after a comprehensive evaluation by investigators from each institution to observe QoL and survival. These patients will be excluded from the evaluation of objective response.

3. Patients with AGC or gastroesophageal junction adenocarcinoma following ≥ second-line chemotherapy failure;

Note: (1) Definition of treatment failure: disease progression during treatment or tumor relapse and metastasis after treatment or intolerable toxicity; (2) for PD, each line of treatment is ≥ 1 cycle or longer with one or more chemotherapy drugs; pilot adjuvant/neoadjuvant therapy is allowed and will be considered first-line systemic chemotherapy for PD in the case of tumor relapse and metastasis during treatment or within 24 weeks after treatment; and (3) upfront therapy can be chemotherapy combined with molecular targeted drugs.

4. Eastern Cooperative Oncology Group (ECOG) performance status (PS) score: 0-2;

5. Normal vital organ functions based on the following standards:

(1) Complete blood count (CBC) (no blood transfusion within 14 days):

a. Hemoglobin (Hb) ≥ 90g/L;

b. Absolute neutrophil count (ANC) ≥ 1.5 × 10^9^/L; and

c. Platelet (PLT) count ≥ 80 × 10^9^/L.

(2) Serum biochemistry:

a. Bilirubin (BIL) < 1.5× upper limit of normal (ULN);

b. Alanine aminotransferase (ALT) and aspartate aminotransferase (AST) < 2.5× ULN; or < 5× ULN in the case of liver metastasis;

c. Serum creatinine (Cr) ≤ 1× ULN, endogenous creatinine clearance ≥ 50 ml/min (Cockcroft-Gault formula);

6. Expected survival ≥ 3 months;

7. The treating physician planned to use apatinib mesylate tablets for treatment;

8. Voluntary participation, with a signed informed consent form (ICF); and

9. Women with childbearing potential must have a negative pregnancy test (serum or urine) within 7 days of enrollment and be willing to use appropriate birth control throughout the observation period and for 8 weeks after the last dose of apatinib mesylate tablets. Men must be surgically sterile or agree to use appropriate birth control throughout the observation period and for 8 weeks after the last dose of apatinib mesylate tablets.

**4.2.2 Exclusion criteria**

1. Uncontrolled hypertension despite treatment with antihypertensive drugs (systolic blood pressure > 140 mmHg/diastolic blood pressure > 90 mmHg), grade ≥ Ⅱ coronary heart disease, arrhythmia (including corrected QT (QTc) prolongation > 450 ms in men or > 470 ms in women), or cardiac insufficiency;

2. Presence of several factors that may affect the absorption of oral drugs (such as swallow difficulty, nausea and vomiting, chronic diarrhea, and intestinal obstruction);

3. Important: patients at risk of gastrointestinal bleeding may not participate, including those with (1) active digestive ulcers, with fecal occult blood (++); (2) a history of melena and hematemesis within 3 months; and (3) fecal occult blood (+) and an unresected primary gastric tumor, if endoscopy indicates ulcerative GC with the possibility of lesion bleeding in the opinion of the treating physician;

4. Coagulopathy (international normalized ratio (INR) > 1.5× ULN, activated partial thromboplastin time (APTT) > 1.5 × ULN), with tendency to bleed;

5. Symptomatic central nervous system metastasis;

6. Pregnant or nursing women; and

7. Any patient deemed unsuitable for this study, as deemed by the treating physician.

**4.2.3 Drop-out/exclusion criteria**

1. Failure to take the drug per protocol, resulting in exclusion from effectiveness and/or safety evaluations; and

2. Major protocol deviation: Use of chemotherapy drugs and/or herbal preparations indicated for GC while in the study.

**4.2.4 Termination criteria:**

1. Subjects who withdraw informed consent and request early exit;

2. Medical imaging indicates disease progression;

3. Becoming pregnant during the study;

4. Intolerable toxicity after two dose adjustments; and

5. Other circumstances that necessitate study exit, as deemed by the investigators.

**4.3 Enrollment**

Competitive enrollment across the sites.

**5. Treatment Prescription**

Treatment is prescribed by the treating physician. Patients are selected for treatment with apatinib mesylate tablets in accordance with the entry criteria. Participation is voluntary.

See apatinib mesylate tablets package insert for prescribing information.

**6. Study Procedures and Data Collection**

**6.1 Data collection plan**

Safety data will be collected from the date of signing the ICF through 30 days after the last dose.

All AEs are recorded on a case report form (CRF) from the date of signing the ICF through the end of the study.

The incidence and severity of AEs will be monitored and evaluated in accordance with NCI CTCAE 4.0.

Each patient is expected to attend all scheduled visits so as to record specific data at different time points during the visits.

**6.2 Screening visit**

The screening visit should be completed within 14 days before treatment cycle 1 and will include electrocardiography (ECG) and chest and abdomen CT or magnetic resonance imaging (MRI). Within 7 days before treatment, a medical history will be obtained, and tests, including ECOG PS score, physical examination, vital signs, CBC, urinalysis, stool test, gastroscopy (as needed), echocardiogram, liver and kidney function, blood electrolytes, coagulation, tumor marker (carcinoembryonic antigen (CEA)), pregnancy test (as needed), and QoL assessment, will be conducted.

**6.3 Visits during the treatment period (cycles 1-3)**

During the treatment period (cycles 1-3), blood pressure will be measured three times per week during the first 2 weeks of treatment and then twice per week thereafter; CBC, urinalysis, and stool tests will be performed every 2 weeks; liver and kidney function and electrolytes will be measured every 4 weeks. ECOG PS score, physical examination, coagulation (prothrombin time (PT), APTT, thrombin time (TT), fibrinogen (Fbg), and ECG will be recorded during each cycle. AEs will be monitored and recorded as they occur.

Drug safety and objective response, QoL score, and CEA will be evaluated at the end of cycles 2 and 3.

**6.4 Additional visits**

Beginning with cycle 4, blood pressure will be measured at least once per week; CBC, urinalysis, stool tests, liver and kidney function, and electrolytes will be measured every four weeks. ECOG PS score, physical examination, coagulation (PT, APTT, TT, Fbg), and ECG will be recorded during each cycle. AEs will be monitored and recorded as they occur.

Drug safety and objective response, QoL score, and CEA will be evaluated every 2 cycles and at the end of treatment (if > 2 weeks since the last imaging date).

**6.5 Follow-up visits**

Patients will enter the posttreatment follow-up period after the last dose of apatinib. Patients who discontinue treatment due to PD should be followed up for survival. Patients who discontinue treatment or withdraw from the study due to reasons other than PD will undergo tumor evaluations every eight weeks until PD, death, the end of the study, or intolerable toxicity. Patients who have no significant adverse reactions after disease progression and are willing to continue apatinib treatment will be closely monitored and followed up for survival.

The following important parameters should be closely monitored and recorded during the follow-up period: time to PD, time of death (phone follow-up is acceptable; a record should be maintained), and other treatments and remedies.

**6.6 Follow-up of adverse events**

Patients will be closely followed up after the last dose for any new AE. Any unresolved AE at the time of apatinib discontinuation will be followed up until it is resolved, has returned to the baseline level, or is no longer clinically relevant.

**6.7 Procedures for patients who discontinue follow-up**

Patients may withdraw informed consent at any time and for any reason. Study withdrawal is considered only if the patient declines follow-ups to monitor his/her survival; otherwise, the patient is still considered in the study. The investigators will make every effort to contact the patients regularly to check their health status, at the very least survival.

**7. Data Management**

**7.1 Data entry and modification**

Data entry and management will be the responsibility of an independent data management agency. The clinical coordinator will use HRTAU-EDC software for clinical data entry and management to ensure data accuracy and timely entry. In the case of any question about the data in the database, the data administrator, medical manager, and study monitor may send an inquiry via the system to the investigator, who will promptly review and answer any questions.

**7.2 Database security**

After data review and verification, the PIs, sponsor, and statisticians will lock the database. No modification to data files will be allowed after locking.

**8. Safety Evaluation**

**8.1 Monitoring adverse events**

An AE is defined as any untoward medical event that occurs after the subject or participant receives a drug or treatment regimen, regardless of the causal relationship to the treatment.

AEs may be any unpleasant signs (including abnormal laboratory tests), symptom, or illness with a temporal relationship or unrelated to the use of apatinib, regardless of whether they are considered related to apatinib.

According to regulations, untoward events that occur before or after treatment are also considered AEs. Therefore, safety monitoring of AEs and SAEs will begin from study entry (signing the ICF) through the end-of-study visit.

**8.2 Rating adverse events**

AEs will be rated as 0-5 in accordance with NCI CTCAE 4.0 (see Annex 2). Any AE not listed in NCI CTCAE 4.0 will be rated based on the following criteria:

Grade I (mild): discomfort, with no effect on daily activities;

Grade Ⅱ (moderate): discomfort that reduces or affects daily activities;

Grade Ⅲ (severe): unable to work or engage in daily activities; and

Grade IV (life-threatening): disability or death.

**8.3 Recording adverse events**

The name, severity, time of onset, duration, treatment measures, and outcomes of each AE that occurs during the study will be recorded and truthfully reported on a CRF. Abnormal laboratory tests will be recorded on a CRF, repeated at least once per week, and followed up until resolution or the end of the study.

Any AEs that occur within 30 days after the last dose will be reported and recorded.

**8.4 Determining the relationship between adverse events and apatinib mesylate tablets**

The correlation between any AE and apatinib will be classified as “definitely”, “probably”, “possibly”, “unlikely”, and “unrelated” (see Table 1). The first three categories indicate that an AE is related to apatinib and will be included as the numerator when calculating the incidence of AE. All subjects will be included as the denominator for safety evaluations.

Table 1. Criteria for Determining the Relationship between Adverse Events and Apatinib Mesylate Tablets

| Criteria | Definitely | Probably | Possibly | Unlikely | Unrelated |
| --- | --- | --- | --- | --- | --- |
| Reasonable temporal relationship | Yes | Yes | Yes | Yes | No |
| Known drug reaction | Yes | Yes | Yes | No | No |
| Improvement after discontinuation | Yes | Yes | Yes or No | Yes or No | No |
| Recurrence after re-administration | Yes | ? | ? | ? | No |
| Additional possible explanations | No | No | No | Yes | Yes |

**8.5 Serious adverse events**

(1) Definition of SAEs

SAEs refer to medical events that occur during a clinical study that require hospitalization or prolonged hospitalization, result in disability, affect participants’ ability to work, are life-threatening or fatal, or lead to congenital malformations. Major unexpected medical events include the following:

⚫ Events leading to death;

⚫ Life-threatening events (defined as risk of death at the time of onset);

⚫ Events that require hospitalization or prolonged hospitalization;

⚫ Events that can lead to permanent or severe disability/dysfunction; and

⚫ Congenital abnormalities or birth defects.

(2) Pregnancy

Pregnancy that occurs during the clinical study will be reported as an SAE.

(3) Disease progression

Disease progression (including progressive signs and symptoms) will not be reported as an SAE. However, death due to disease progression during the study or safety reporting period will be reported as an SAE. Hospitalization due to progressive signs and symptoms will not be reported as an SAE. If cancer patients die during the study or safety reporting period, the event leading to death will be reported as an SAE.

(4) Other anticancer treatments

If the subject starts to receive other anticancer treatments, non-fatal AEs will be reported as of the start of new anticancer treatments. If death occurs during the SAE reporting period after the end of study treatment, SAEs will be reported regardless of whether the patient receives other treatments.

(5) Hospitalization

All AEs that result in hospitalization or prolonged hospitalization during the clinical study will be considered SAEs. Any initial admission (even if it is less than 24 hours) will be considered hospitalization.

Hospitalization does not include the following:

⚫ Rehabilitation facilities or nursing homes;

⚫ Regular emergency room admission; and

⚫ Same-day surgery (such as outpatient/same-day/ambulatory surgery).

Hospitalization or prolonged hospitalization unrelated to worsening AEs will not be considered SAEs:

⚫ Admission due to a preexisting condition, without any new AE or worsening of the preexisting condition (such as to check preexisting, ongoing laboratory abnormalities before the study);

⚫ Hospitalization due to administration or insurance (such as annual routine check-up);

⚫ Hospitalization specified in the study protocol (such as procedures specified in the protocol);

⚫ Elective hospitalization unrelated to worsening of an AE (such as elective cosmetic surgery);

⚫ Scheduled treatment or surgery will be recorded in the protocol and/or the subject’s baseline data; and

⚫ Admission to receive blood products.

Diagnostic or therapeutic invasive (such as surgery) and noninvasive procedures will not be reported as AEs. However, any condition that results in a procedure will be reported if it meets the criteria for AEs. For example, acute appendicitis that occurs during the AE reporting period will be reported as an AE, but appendectomy will be recorded as the treatment of this AE.

(6) SAE reporting procedures

The SAE reporting period will be from signing the ICF through 30 calendar days after the last dose of apatinib mesylate tablets (including the 30^th^ day). During the study, any SAE will be reported to the clinical monitor and PIs within 24 hours and reported on the New Drug Clinical Study Serious Adverse Event (SAE) Reporting Form (signed and dated), which will be promptly faxed to the sponsor, lead site(s), Ethics Committee of clinical sites, NMPA, and the investigator’s regional (provincial or metropolitan) FDA.

During continued treatment after the end of the study, any SAE will be reported to the sponsor within 24 hours. All SAEs will be recorded on an SAE reporting form. All SAEs that occur from the start of continued treatment through 30 days after the last dose will be reported. SAES that occur after the 30-day period will not generally be reported unless they are suspected to be related to apatinib mesylate tablets.

For SAEs, the symptoms, severity, time of onset, time of treatment, measures taken, follow-up time and method, and outcomes will be carefully monitored and recorded. If the investigator believes that an SAE is unrelated to apatinib mesylate tablets but is potentially related to the study conditions (such as discontinuation of previous treatment or comorbidities during the study), the relationship will be explained in an SAE narrative on a CRF. If the intensity of an ongoing SAE or its relationship to apatinib mesylate tablets changes, a follow-up SAE report will promptly sent to the sponsor. All SAEs will followed up until resolution or stabilization.

Note: SAE contacts

| Institution | Contact | Phone | Fax |
| --- | --- | --- | --- |
| NMPA | Safety Monitoring Committee | 010-68313344-1003 | 010-88363228 |
| Fudan University Shanghai Cancer Center | Ethics Committee | 021-64175590-88503 | 021-64036901 |
| PLA 81 Hospital | Drug Clinical Trial Center | 025-84453667 | 025-84453906 |
| Hengrui Pharmaceuticals Co., Ltd. | Medical Affairs | 021-68868570/71*832 | 021-50819731 |

**9. Statistical Processing**

**9.1 Analysis populations**

Safety set (SS): safety data set that will include all enrolled subjects who have received at least one dose of apatinib mesylate tablets and have at least one safety evaluation;

FAS: an intent-to-treat (ITT) data set that will include all randomized subjects who have received at least one dose of the apatinib mesylate tablets; subjects who did not receive any treatment after randomization and who do not meet inclusion criterion 2 will be excluded from FAS; and

Per-protocol set: a subset of FAS that will include all per-protocol, compliant subjects who did not receive any other GC treatment during the study and who have complete CFR data. Missing data will not be imputed.

**9.2 Analysis variables**

**9.2.1 Primary measures**

AEs will be coded according to MedDRA, and each AE will be assigned a system organ classification (SOC) and a preferred term. The severity of AEs will be evaluated in accordance with NCI CTCAE 4.0.

The primary endpoints will include the incidence and severity of AEs.

**9.2.2 Secondary measures**

**a. Safety measures**

⚫ The incidence of treatment-emergent AEs

⚫ The incidence of AEs leading to treatment discontinuation and/or early withdrawal

⚫ The incidence of SAEs and drug-related SAEs

⚫ Mortality

⚫ Drug discontinuation due to AEs

⚫ Changes in vital signs after treatment (heart rate, blood pressure, temperature, respiration)

⚫ Changes in physical examination results after treatment

⚫ Clinical laboratory abnormalities

**b. Efficacy measures:**

⚫ OS, defined as the time from study entry (signing the ICF) to all-cause death. For subjects who are alive at the last follow-up, the last follow-up date is censored for OS.

⚫ PFS, defined as the time from study entry (signing the ICF) to tumor progression or all-cause death. For subjects without tumor progression or death, the last valid tumor evaluation date is censored for PFS.

⚫ ORR: CR + PR

⚫ DCR: CR + PR + SD

Note: The RECIST criteria are used mainly for the observation and evaluation of objective responses to cytotoxic chemotherapy drugs and cannot comprehensively or accurately reflect the efficacy of molecular targeted drugs. Apatinib mesylate tablets may be continued, under close monitoring, if medical imaging indicates significant degeneration, necrosis, or liquefaction in the lesion even though the lesion has grown or if clinical symptoms and/or QoL improve, the treating physician believes that treatment continuation will bring survival benefits, and the patient has no significant adverse reactions and is willing to continue treatment.

**9.3 Statistical methods**

**9.3.1 General principles:**

Biostatisticians and PIs will work together to develop a statistical analysis plan based on the study protocol and finalize the plan before the database is locked.

SAS v9.2 will be used, and 95% confidence intervals (CIs) will be provided.

For continuous variables, the number of subjects, mean, standard deviation, median, minimum, and maximum will be provided. For categorical variables, frequency and percent will be tabulated.

**9.3.2 Primary analyses**

The primary endpoints will include the incidence, severity, correlation, risk factors, measures taken, and outcomes of each AE.

⚫ Study period, drug exposure

SS will be used as the study population to describe the dosing rate, actual dose intensity, and treatment discontinuation and the cause.

⚫AEs

AEs will mainly be described with descriptive statistics. Laboratory tests that are normal before treatment but abnormal after treatment or abnormal before treatment and worsen after treatment will be described, and their relation to apatinib mesylate tablets will be indicated.

All other AEs will be listed per subject. Treatment-emergent SAEs, death, and the incidence and 95% CIs for drug discontinuation due to an AE will be calculated. AEs will be summarized per SOC and preferred terms.

The incidence, severity, correlation, risk factors, measures taken, and outcomes of important known AEs (hypertension, bleeding, proteinuria/renal toxicity, and HFS) will be monitored and recorded.

The incidence, severity, correlation, risk factors, measures taken, and outcomes of important potential AEs (grade 3/4 diarrhea, gastrointestinal fistula/perforation, wound healing complications, decreased left ventricular ejection fraction/heart failure, thrombosis/embolization, liver toxicity, hypothyroidism) will be monitored and recorded.

**9.3.3 Secondary analyses**

⚫ **Effectiveness**

The Kaplan-Meier method will be used to plot OS and PFS curves. The mOS and mPFS and their 95% CIs will be estimated.

ORR and DCR and their 95% CIs will be calculated.

⚫ **Concomitant medications (during the study)**

Concomitant medications during the study will be summarized, including any change in concomitant medications during the screening period, new concomitant medications after screening, and drugs used to treat any AE, as well as usage frequency of each drug.

**9.4 Determination of sample size**

Number of subjects: n = 2000

**10. Quality Control and Quality Assurance**

All personnel involved in this study, including clinicians and monitors, will receive proper training. They also acknowledge that they will conduct the study according to the protocol, protect the safety and rights of subjects, follow standard operating procedures, and accurately record and report all study data.

**11. Ethics, Regulations, Administrative Principles**

**11.1 Ethics principles**

This study will be conducted in accordance with the principles established by the 18th World Medical Association General Assembly (Helsinki, 1964) and all subsequent amendments.

**11.2 Laws and regulations**

This study will comply with national laws and regulations and current NMPA-issued Good Clinical Practice (GCP) guidelines.

**11.3 Data protection**

The database of the sponsor, Hengrui Pharmaceuticals Co., Ltd., may contain personal data of patients and investigators; these data will be processed in accordance with national laws and regulations. When archiving or processing personal data related to investigators and/or patients, care will be taken to protect personal privacy. Hengrui Pharmaceuticals Co., Ltd. shall take all appropriate measures to protect these data and prevent any unauthorized third-party access to these data.

**11.4 Confidentiality agreement**

All information, materials, and unpublished documents provided to the investigators by the sponsor will not be disclosed to any other individual or institution without written permission of Hengrui Pharmaceuticals Co., Ltd.

**11.5 Record maintenance**

The investigators will properly maintain study documents until 5 years after the end of the study. For patient records, the investigators will follow the regulations of national health authorities.

**11.6 Sponsor audit and regulatory inspection**

The site will allow the sponsor’s auditor/regulatory inspectors to access and review subjects’ study records. These personnel are bound by the principles of professional confidentiality and will not disclose any patient’s personal identity or medical information.

The sites agree to make every effort to assist in audits and inspections and allow auditors/inspectors to access all necessary equipment, date, and files and take corrective measures to address any issue discovered during the audit or inspection process.

**12. Protocol Amendment**

Any protocol amendment will be approved by the Science Committee and signed by the PI and the sponsor. Major changes will be submitted to the Ethics Committee of the lead site for review and approval. A written amendment will be prepared.

**13. Use of Documents and Study Results**

**13.1 Ownership and use of study data and study results**

The sponsor will own the study data and information. The Science Committee will have full access to the final data to conduct scientific analyses of the study results, reach conclusions, and publish reports. Any other individual or institution will not be allowed to use unpublished study data without permission from the sponsor, Hengrui Pharmaceuticals Co., Ltd.

**13.2 Publications**

All sites and investigators involved in this study agree to authorize the Science Committee to first release and/or publish study results. The signatures and the order of names on publications will be based on individual contributions to the study, including protocol development, participation in discussions, and the quantity and quality of enrollment. Before initial publication, any other individual or institution may not publish without authorization. For any subsequent release or publication, individuals involved in the study, including sub-studies, must consult with the Science Committee and the sponsor and reference this study and initial publication. After prior notification to the sponsor (for internal review and comments), the Science Committee will make the final decision about any abstract/bulletin/article. The sponsor may request to list or not to list the sponsor and/or its personnel in the article.

**14. References**

[1] De Martel C, Forman D, Plummer M. Gastric cancer: epidemiology and risk factors [J]. Gastroenterol Clin N Am, 2013, 42(2):219-240.

[2] Zheng RS, Zhang SW, Wu LY, et al. Report of incidence and mortality from China cancer registries in 2008 [J]. China Cancer, 2012, 21(1): 1-12

[3] Yu S, Liu TS. Progress of molecular targeted therapy for gastric cancer [J]. Chinese Clinical Oncology, 2014, 19(1): 1-8.

[4] Jemal A, Bray F, Center MM, et al. Global cancer statistics [J]. CA Cancer J Clin, 2011, 61(2):69-90.

[5] Qin SK, Gong XL. Chemotherapy for advanced gastric carcinoma: current treatments and prospects [J]. Chinese Clinical Oncology, 2006, 11(9): 641-652.

[6] Zhang W, Wang JW. Epidemiology and classification of gastric cancer [J]. Chinese General Practice, 2010, 13(11): 16-17.

[7] Wagner AD, Grothe W, Haerting J, et al. Chemotherapy in advanced gastric cancer: a systematic review and meta-analysis based on aggregate data [J]. J Clin Oncol, 2006, 24(18): 2903-2909.

[8] Cunningham D, Starling N, Rao S, et al. Capecitabine and oxaliplatin for advanced esophagogastric cancer [J]. N Engl J Med, 2008, 358(1): 36-46.

[9] Van Cutsem E, Moiseyenko VM, Tjulandin S, et al. Phase Ⅲ study of docetaxel and cisplatin plus fluorouracil compared with cisplatin and fluorouracil as first-line therapy for advanced gastric cancer: a report of the V325 Study Group [J]. J Clin Oncol, 2006, 24(31): 4991-4997.

[10] Van CUTSEM E, de HAAS S, KANG Y K, et al. Bevacizumab in combination with chemotherapy as first-line therapy in advanced gastric cancer: a biomarker evaluation from the AVAGAST randomized phase Ⅲ trial [J]. J Clin Oncol, 2012, 30(17): 2119-2127.

[11] Bang YJ, Kang YK, Kang WK, et al. Phase II study of sunitinib as second-line treatment for advanced gastric cancer. Invest New Drugs. 2011, 29(6) L1449-1458.

[12] Moehler M, Mueller A, Hartmann JT, et al. An open-label, multi-centre biomarker-oriented AIO phase II trial of sunitinib for patients with chemo-refractory advanced gastric cancer [J]. Eur J Cancer, 2011, 47(10): 1511-1520.

[13] YI J H, LEE J, LEE J, et al. Randomised phase Ⅱ trial of docetaxel and sunitinib in patients with metastatic gastric cancer who were previously treated with fluoropyrimidine and platinum [J]. Br J Cancer, 2012, 106(9): 1469-1474.

[14] SUN W, POWELL M, O' DWYER P J, et al. Phase Ⅱ study of sorafenib in combination with docetaxel and cisplatin in the treatment of metastatic or advanced gastric and gastroesophageal junction adenocarcinoma: ECOG 5203 [J]. J Clin Oncol, 2010, 28(18): 2947-2951.

[15] Kim C, Lee JL, Choi YH, et al. Phase I dose-finding study of sorafenib in combination with capecitabine and cisplatin as a first-line treat­ment in patients with advanced gastric cancer[J]. Invest New Drugs. 2012, 30(1): 306-315.

[16] Qin S. Phase III study of apatinib in advanced gastric cancer: A randomized, double-blind, placebo-controlled trial. J Clin Oncol 32:5s, 2014 (suppl; abstr 4003).

[17] Li J, Qin SK, Xu JM, et al. Apatinib for chemotherapy-refractory advanced metastatic gastric cancer: results from a randomized, placebo-controlled, parallel-arm, phase Ⅱ trial [J]. J Clin Oncol, 2013, 31(26): 3219-3225.

[18]. Fuchs, C.S, Tomasek J, et al. Ramucirumab monotherapy for previously treated advanced gastric or gastro-oesophageal junction adenocarcinoma (REGARD): an international, randomised, multicentre, placebo-controlled, phase 3 trial[J]. Lancet, 2014. 383(9911): 31-
